# Multimodal subspace independent vector analysis effectively captures the latent relationships between brain structure and function

**DOI:** 10.1101/2023.09.17.558092

**Published:** 2024-10-22

**Authors:** Xinhui Li, Peter Kochunov, Tulay Adali, Rogers F. Silva, Vince D. Calhoun

**Affiliations:** 1 Tri-institutional Center for Translational Research in Neuroimaging and Data Science, Georgia State University, Georgia Institute of Technology, Emory University, Atlanta, GA, USA; 2 School of Electrical and Computer Engineering, Georgia Institute of Technology, Atlanta, GA, USA; 3 Maryland Psychiatric Research Center, Department of Psychiatry, School of Medicine, University of Maryland, Baltimore, MD, USA; 4 Department of Computer Science and Electrical Engineering, University of Maryland Baltimore County, Baltimore, MD, USA

**Keywords:** multimodal fusion, latent variable models, structural and functional MRI, age, sex, schizophrenia

## Abstract

A key challenge in neuroscience is to understand the structural and functional relationships of the brain from high-dimensional, multimodal neuroimaging data. While conventional multivariate approaches often simplify statistical assumptions and estimate one-dimensional independent sources shared across modalities, the relationships between true latent sources are likely more complex – statistical dependence may exist within and between modalities, and span one or more dimensions. Here we present Multimodal Subspace Independent Vector Analysis (MSIVA), a methodology to capture both joint and unique vector sources from multiple data modalities by defining both cross-modal and unimodal subspaces with variable dimensions. In particular, MSIVA enables flexible estimation of varying-size independent subspaces within modalities and their one-to-one linkage to corresponding subspaces across modalities. As we demonstrate, a main benefit of MSIVA is the ability to capture subject-level variability at the voxel level within independent subspaces, contrasting with the rigidity of traditional methods that share the same independent components across subjects. We compared MSIVA to a unimodal initialization baseline and a multimodal initialization baseline, and evaluated all three approaches with five candidate subspace structures on both synthetic and neuroimaging datasets. We show that MSIVA successfully identified the ground-truth subspace structures in multiple synthetic datasets, while the multimodal baseline failed to detect high-dimensional subspaces. We then demonstrate that MSIVA better detected the latent subspace structure in two large multimodal neuroimaging datasets including structural MRI (sMRI) and functional MRI (fMRI), compared with the unimodal baseline. From subsequent subspace-specific canonical correlation analysis, brain-phenotype prediction, and voxelwise brain-age delta analysis, our findings suggest that the estimated sources from MSIVA with optimal subspace structure are strongly associated with various phenotype variables, including age, sex, schizophrenia, lifestyle factors, and cognitive functions. Further, we identified modality- and group-specific brain regions related to multiple phenotype measures such as age (e.g., cerebellum, precentral gyrus, and cingulate gyrus in sMRI; occipital lobe and superior frontal gyrus in fMRI), sex (e.g., cerebellum in sMRI, frontal lobe in fMRI, and precuneus in both sMRI and fMRI), schizophrenia (e.g., cerebellum, temporal pole, and frontal operculum cortex in sMRI; occipital pole, lingual gyrus, and precuneus in fMRI), shedding light on phenotypic and neuropsychiatric biomarkers of linked brain structure and function.

## 1 Introduction

Neuroimaging techniques such as magnetic resonance imaging (MRI) have been developed to understand the structural and functional properties of the brain, as well as their relationships to behavior. However, it is challenging to directly associate behavior measures with raw MRI data, which typically includes tens of thousands of voxels and subjects. Although the data in its original space appears complex, its intrinsic dimensionality can be significantly lower. Recent studies have found that neural representations in low-dimensional subspaces form the basis that supports motor functions such as reaching (Churchland et al., 2012; Pandarinath et al., 2018) and timing (Remington et al., 2018; Wang et al., 2018), and cognitive functions such as perception (Bao et al., 2020; Chang & Tsao, 2017; Semedo et al., 2019; She et al., 2024), generalization (Bernardi et al., 2020; Boyle et al., 2024; Courellis et al., 2024), and decision-making (Hajnal et al., 2024; Johnston et al., 2024). Hence, it is important to develop latent variable models to learn low-dimensional representations and structures from high-dimensional data. In addition, each neuroimaging modality has its own strengths and weaknesses, and only captures limited information about the brain. For example, structural MRI (sMRI) provides high-resolution anatomical structure of the brain but does not capture temporal dynamics, while functional MRI (fMRI) measures blood-oxygenation-level-dependent (BOLD) signals over time at the cost of lower spatial resolution. Joint analysis of sMRI and FMRI can offer rich spatio-temporal information in the brain that is not captured by a single modality. With the increasing availability of multimodal neuroimaging datasets, it is necessary to develop multivariate approaches to effectively capture interpretable and multifaceted information about the brain and its disorders from multiple imaging modalities (Calhoun & Sui, 2016; Lahat et al., 2015; Sui et al., 2012; Zhang et al., 2020).

A variety of data-driven multivariate approaches have been developed to jointly analyze multiple neuroimaging datasets or data modalities, including joint independent component analysis (jICA) (Calhoun & Adali, 2008; Calhoun, Adali, Giuliani, et al., 2006; Calhoun, Adali, Pearlson, & Kiehl, 2006; Franco et al., 2008), linked ICA (Groves et al., 2011), multimodal canonical correlation analysis (mCCA) (Correa et al., 2008, 2010; Mohammadi-Nejad et al., 2017), jICA+mCCA (Sui et al., 2011, 2013), and independent vector analysis (IVA) (Adali et al., 2015a, 2015b). Notably, a unified framework Multidataset Independent Subspace Analysis (MISA) (Silva et al., 2020) has recently been introduced, encompassing multiple latent variable models, such as ICA (Comon, 1994), IVA (Adali et al., 2014; Kim et al., 2006), and independent subspace analysis (ISA) (Cardoso, 1998). MISA can be applied to identify latent sources from multiple neuroimaging modalities, including sMRI and fMRI (Silva et al., 2020). More recently, a multimodal IVA (MMIVA) fusion method built upon MISA has been proposed to identify linked biomarkers related to age, sex, cognition, and psychosis in two large multimodal neuroimaging datasets (Silva et al., 2021). However, one limitation of many existing approaches including MMIVA is that they assume that sources are one-dimensional and independent within each modality, i.e. the subspace structure is an identity matrix. The underlying relationships between true latent sources are likely more complex – statistical dependence may exist within and across modalities, and span one or more dimensions. For example, sources from the same modality may be linked, potentially grouped by their anatomical or functional properties, and thus would not be optimally captured by MMIVA.

Aiming to better detect the statistical relationships from multimodal data, we present a novel methodology, Multimodal Subspace Independent Vector Analysis (MSIVA), that captures linkage of vector sources by defining cross-modal and unimodal subspaces with variable dimensions (Li et al., 2023). MSIVA is built upon MMIVA by defining a block diagonal matrix as the subspace structure, instead of the identity matrix used in MMIVA. In addition, MSIVA is initialized with the weight matrices obtained by combining multimodal group principal component analysis (MGPCA) across modalities with separate ICAs for each modality. By design, MSIVA can simultaneously estimate two types of latent sources – those linked across all modalities and those unique to a specific modality, as well as their underlying relationships. Moreover, by leveraging higher-dimensional subspaces, MSIVA sources show greater representation power, which supports downstream analyses at both individual and voxel levels.

To comprehensively evaluate the effectiveness of MSIVA, we compared MSIVA with a fully unimodal initialization approach and a fully multimodal initialization approach. We first simulated multiple synthetic datasets to evaluate whether MSIVA can successfully reconstruct both joint and unique sources, as well as the ground-truth subspace structures. Next, we applied MSIVA and the baseline approach to two large multimodal neuroimaging datasets, the UK Biobank dataset (Miller et al., 2016) and a schizophrenia (SZ) patient dataset combined from several studies (Aine et al., 2017; Keator et al., 2016; Tamminga et al., 2014). Our results indicate that MSIVA better detected the latent subspace structures in the neuroimaging datasets compared with the baseline approach. Using CCA (Hotelling, 1992), we conducted a follow-up assessment of each cross-modal subspace separately and identified projections within the optimal subspace structure yielding the post-CCA linked sources. We then performed age regression, sex classification, and diagnosis classification to investigate the associations between these linked sources and phenotype measures. Results from brain-phenotype modeling suggest that the post-CCA sources are associated with age, sex and SZ-related effects. Furthermore, we proposed a voxelwise brain-age delta analysis using reconstructed data from MSIVA. We found that brain-age gap can be explained by several phenotype measures, such as lifestyle factors and cognitive test scores. Lastly, we identified modality- and group-specific brain regions related to age, sex, SZ, cognitive function, and physical exercise. Overall, our findings suggest that MSIVA can effectively reveal the latent sources related to phenotype variables from multimodal neuroimaging data, thereby uncovering linked phenotypic and neuropsychiatric biomarkers of brain structure and function.

## 2 Methods

### 2.1 Multimodal subspace independent vector analysis

We consider the following problem that each observed data modality is a linear mixture of latent sources:

(1) $$X^{\left[m\right]}=A^{\left[m\right]}S^{\left[m\right]},$$

where $X^{\left[m\right]}\in R^{V\times N}$ is the observed data, $A^{\left[m\right]}\in R^{V\times C}$ is a linear mixing matrix, $S^{\left[m\right]}\in R^{C\times N}$ is the latent source, m is the modality index, V is the input feature dimensionality, and N is the number of samples. Sources across M modalities are either statistically dependent or independent, according to the subspace structure S defined using available a priori information. We aim to recover the latent sources ${\overset{ˆ}{S}}^{\left[m\right]}\in R^{C\times N}$ by estimating a linear unmixing matrix $W^{\left[m\right]}\in R^{C\times V}$:

(2) $${\overset{ˆ}{S}}^{\left[m\right]}=W^{\left[m\right]}X^{\left[m\right]}.$$

**Figure 1:**
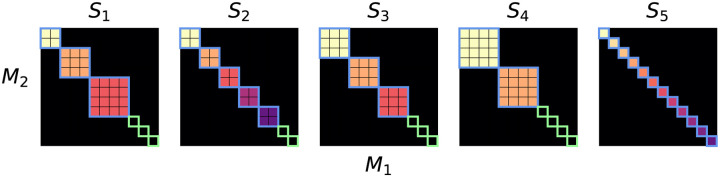
Five plausible candidate subspace structures ($S_{1}-S_{5}$) for two modalities ($M_{1}-M_{2}$). Each panel depicts the idealized association between sources from two modalities $\left(M_{1}-M_{2}\right)$, across five different plausible scenarios $\left(S_{1}-S_{5}\right)$. The size of each block represents the number of sources within a subspace (the subspace size). The colorful subspaces highlighted in blue are linked between modalities, whereas the black subspaces highlighted in green (1 × 1 blocks in $S_{1}-S_{4}$) are specific to each modality (no cross-modal correlation). For each modality, sources within the same subspace are statistically dependent while sources in different subspaces are statistically independent.

**Figure 2:**
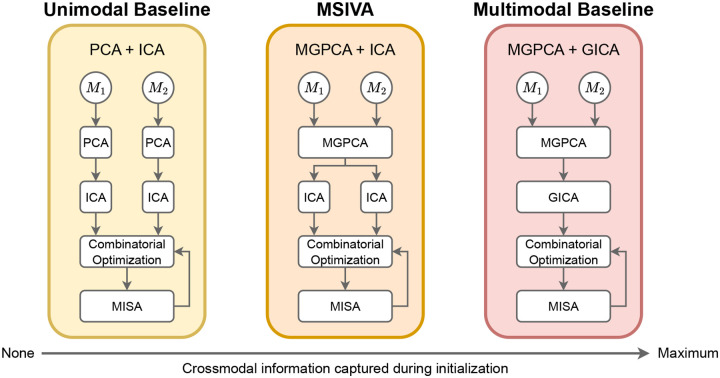
Overview of three proposed initialization workflows. The initialization approaches from left to right are separate PCAs followed by separate ICAs (PCA + ICA); multimodal group PCA with separate ICAs per modality (MGPCA + ICA); multimodal group PCA with group ICA (MGPCA + GICA). The MGPCA + ICA initialization workflow is denoted as MSIVA. After initialization, the combinatorial optimization and numerical optimization with the MISA loss were performed for sufficient iterations until the loss value converged.

We refer to our proposed approach as Multimodal Subspace Independent Vector Analysis (MSIVA) because it is an extension of MMIVA by allowing higher-dimensional cross-modal subspaces that are constrained to have the same size across modalities. We consider five candidate subspace structures that define different types of multimodal relationships (Figure 1) and three initialization workflows that capture different amounts of joint information (Figure 2). Given a candidate subspace structure, MSIVA consists of iterative combinatorial optimization of the source estimates (cross-modal subspace alignment) and numerical optimization of the MISA loss (Equation 5). This process is repeated for each of the five candidate subspace structures, followed by a best-fit determination based on the final quantitative metrics of all candidates.

#### 2.1.1 Subspace structures

Our interest lies in identifying groups of linked (i.e. *not* independent) sources within each modality, while assuming sources in different groups are statistically independent. Here, these source groups are referred to as *subspaces*. In addition, we aim to detect cross-modal linkage (i.e. statistical dependence) between subspaces. This requires solving a challenging combinatorial optimization problem. For simplicity, we limit the search space of cross-modal linkage by assuming that statistical dependence occurs only between higher-dimensional (two-dimensional or above) subspaces with the same size across modalities. Additionally, we assume all modality-specific subspaces to be one-dimensional (1D), i.e. a single source.

Building on the MISA framework, we require a user-defined candidate subspace structure that specifies the expected linkage pattern. The goal of MSIVA is to determine which one of the candidate subspace structures best fits the observed data. Two to four dimensions are commonly used to cluster functional networks in functional imaging literature (Ma et al., 2010, 2011). Thus, we proposed five plausible subspace structures $\left(S_{1}-S_{5}\right)$ in two modalities $\left(M_{1}-M_{2}\right)$, all with 12 sources in each modality (Figure 1):

- $S_{1}$: One two-dimensional (2D) cross-modal subspace, one three-dimensional (3D) cross-modal subspace, one four-dimensional (4D) cross-modal subspace, and three 1D unimodal subspaces.
- $S_{2}$: Five 2D cross-modal subspaces and two 1D unimodal subspaces.
- $S_{3}$: Three 3D cross-modal subspaces and three 1D unimodal subspaces.
- $S_{4}$: Two 4D cross-modal subspaces and four 1D unimodal subspaces.
- $S_{5}$: Twelve 1D cross-modal subspaces (no unimodal subspaces, as in MMIVA).

#### 2.1.2 MSIVA initialization workflow

The MSIVA initialization workflow first utilized multimodal group principal component analysis (MGPCA) to identify common principal components across all modalities and then applied ICA on the MGPCA-reduced data of each modality. Unlike principal component analysis (PCA) that identifies orthogonal directions of maximal variation for each modality separately, MGPCA identifies directions of maximal *common* variation across all modalities. Eigenvectors were computed based on the average of the scaled covariance matrices:

(3) $$\Sigma_{\text{avg}}=\frac{1}{M}\overset{M}{\underset{m=1}{\sum }}N\frac{\Sigma^{\left[m\right]}}{\text{trace}\left(\Sigma^{\left[m\right]}\right)}=\frac{1}{M}\overset{M}{\underset{m=1}{\sum }}N\frac{X^{{\left[m\right]}^{⊤}}X^{\left[m\right]}}{{\left\|X^{\left[m\right]}\right\|}_{\text{Fr}}^{2}},$$

where $\Sigma^{\left[m\right]}=\frac{X^{{\left[m\right]}^{⊤}}X^{\left[m\right]}}{V-1}\approx E\left[X^{{\left[m\right]}^{⊤}}X^{\left[m\right]}\right],E\left[\cdot \right]$ is the expectation operator, and $\|\cdot {\|}_{\text{Fr}}$ indicates the Frobenius norm. The scaling factor $\frac{\text{trace}\left(\Sigma^{\left[m\right]}\right)}{N}$ is the ratio of the variance in the modality to the number of samples. We define the whitening matrix $W_{\text{MGPCA}}^{\left[m\right]}$ as follows:

(4) $$W_{\text{MGPCA}}^{\left[m\right]}=\sqrt{N-1}\Lambda^{-\frac{1}{2}}U^{[m{]}^{⊤}}\lambda^{\left[m\right]},$$

where Λ and Q are the top C eigenvalues and eigenvectors of $\Sigma_{\text{avg}}$, respectively, $U^{\left[m\right]}=\left(\lambda^{\left[m\right]}X^{\left[m\right]}\right)Q\Lambda^{-\frac{1}{2}},\;\lambda^{\left[m\right]}=\sqrt{\frac{N}{M(V-1)\text{trace}\left(\Sigma^{\left[m\right]}\right)}}=\sqrt{\frac{N}{M{\left\|X^{\left[m\right]}\right\|}_{Fr}^{2}}}$.

Next, the MGPCA-reduced data from each modality $X_{\text{r}}^{\left[m\right]}=W_{\text{MGPCA}}^{\left[m\right]}X^{\left[m\right]}$ underwent a separate ICA estimation using the Infomax algorithm (Bell & Sejnowski, 1995) initialized with an identity matrix to obtain C independent sources per modality ${\overset{ˆ}{S}}_{\text{Infomax}}^{\left[m\right]}=W_{\text{Infomax}}^{\left[m\right]}X_{\text{r}}^{\left[m\right]}$. These estimates were further optimized by running MISA as a unimodal ICA model initialized with $W_{\text{Infomax}}^{\left[m\right]}$, leading to the final ICA source estimates ${\overset{ˆ}{S}}_{\text{ICA}}^{\left[m\right]}=W_{\text{ICA}}^{\left[m\right]}X_{\text{r}}^{\left[m\right]}$. Finally, multimodal MISA was initialized by the combined MGPCA+ICA estimates $W_{0}^{\left[m\right]}=W_{\text{ICA}}^{\left[m\right]}W_{\text{MGPCA}}^{\left[m\right]}$ from both modalities. Subsequently, we compared MSIVA with a fully unimodal initialization workflow and a fully multimodal initialization workflow to comprehensively evaluate method performance.

#### 2.1.3 Unimodal initialization workflow

The unimodal initialization workflow simply applied PCA and ICA on each modality separately. We first projected the imaging data matrix from each modality $X^{\left[m\right]}$ into a reduced data matrix $X_{\text{r}}^{\left[m\right]}$ with C principal components and obtained the corresponding whitening matrix $W_{\text{PCA}}^{\left[m\right]}$. Next, we applied ICA on each reduced data matrix $X_{\text{r}}^{\left[m\right]}$ to obtain C independent sources and the corresponding unmixing matrix $W_{\text{ICA}}^{\left[m\right]}$. The MISA initialization matrix in the unimodal baseline was defined as $W_{0}^{\left[m\right]}=W_{\text{ICA}}^{\left[m\right]}W_{\text{PCA}}^{\left[m\right]}$.

#### 2.1.4 Multimodal initialization workflow

The multimodal initialization workflow sequentially applied MGPCA and group ICA (GICA) across all data modalities, resulting in the weight matrices $W_{\text{MGPCA}}^{\left[m\right]}$ and $W_{\text{GICA}}^{\left[m\right]}$. GICA performed ICA on the combined MGPCA-reduced data from all M modalities, i.e. $X_{\text{r}}=\sum_{m=1}^{M}X_{\text{r}}^{\left[m\right]}$. MISA in the multimodal baseline was initialized by $W_{0}^{\left[m\right]}=W_{\text{GICA}}^{\left[m\right]}W_{\text{MGPCA}}^{\left[m\right]}$.

#### 2.1.5 Alternating combinatorial and numerical optimizations

All three workflows utilize MISA’s greedy combinatorial optimization and objective function to estimate latent sources. MISA uses the relative gradient and L-BFGS algorithm (Liu & Nocedal, 1989) in a barriertype optimization (fmincon from MATLAB’s Optimization Toolbox). Greedy combinatorial optimization and MISA optimization were performed iteratively until the loss value converged. Specifically, we ran 10 iterations for synthetic data, and 20 iterations for neuroimaging data. The loss function ℒ(⋅) (Silva et al., 2020) is defined as the Kullback-Leibler (KL) divergence between the joint distribution of all sources $p(\overset{ˆ}{S})$ and the product of all K subspace distributions $q(\overset{ˆ}{S})=\prod_{k=1}^{K}p\left({\overset{ˆ}{S}}_{k}\right)$, which is equivalent to mutual information among K subspaces. The subspace distributions are modeled as the joint Kotz distribution (Kotz, 1975) of the sources within each subspace. Thus, subspaces are assumed to be statistically independent of each other within each modality. Sources within a subspace are considered to be dependent on (or linked to) one another. We want to minimize the loss function ℒ(⋅) by solving the following optimization problem:

(5) $$\text{min}\;ℒ\left(\hat{S}\right)=\text{min}\;E\left[\text{ln}\frac{p\left(\hat{S}\right)}{q\left(\hat{S}\right)}\right]\;=\text{min}\;E\left[\text{ln}\;p\left(\hat{S}\right)\right]-\overset{K}{\underset{k=1}{\sum }}E\left[\text{ln}\;p\left({\hat{S}}_{k}\right)\right]\;=\underset{\underset{k=1,\ldots ,K}{\hat{W},P_{k},}}{\text{min}}\;E\left[\text{ln}\;p\left(\hat{W}X\right)\right]-\overset{K}{\underset{k=1}{\sum }}E\left[\text{ln}\;p\left(P_{k}\hat{W}X\right)\right],$$

where $\overset{ˆ}{S}=\left[{\overset{ˆ}{S}}^{\left[1\right]};\ldots ;{\overset{ˆ}{S}}^{\left[M\right]}\right]\in R^{MC\times N}$^1^ is the estimated sources for all M modalities. $X=\left[X^{\left[1\right]};\ldots ;X^{\left[M\right]}\right]\in R^{MV\times N}$ is the concatenated data with all M modalities. $\overset{ˆ}{W}\in R^{MC\times MV}$ is the estimated block-diagonal unmixing matrix, such that ${\overset{ˆ}{S}}^{\left[m\right]}={\overset{ˆ}{W}}^{\left[m\right]}X^{\left[m\right]}.P_{k}\in R^{C_{k}\times MC}$ is the k-th subspace assignment matrix defined by the subspace structure S in Section 2.1.1, and $C_{k}$ is the number of sources in the kth subspace.

### 2.2 Datasets

#### 2.2.1 Synthetic data

For each subspace structure S, we generated a synthetic dataset with two modalities $X=\left[X^{\left[1\right]};X^{\left[2\right]}\right]\in R^{2V\times N}$, where V is the dimensions of input features (V=20000) and N is the number of samples (N=3000).V and N were chosen to approximate the number of voxels and samples in the UK Biobank neuroimaging dataset (see Section 2.2.2). Each data modality is a linear mixture of 12 sources spanning the subspaces defined in $S,X^{\left[m\right]}=A^{\left[m\right]}S^{\left[m\right]},A^{\left[m\right]}\in R^{V\times C},S^{\left[m\right]}\in R^{C\times N},m\in \{1,2\}$, and C=12. Each subspace is independently sampled from a multivariate Laplace distribution. Hence, the marginal distributions correspond to the different sources within each subspace. Cross-modal sources within each linked subspace are dependent with correlation coefficients uniformly sampled from 0.65 to 0.85. Unimodal sources (1D subspaces in $S_{1}-S_{4}$) are independent from all others, i.e. their correlation coefficient is 0.

#### 2.2.2 Neuroimaging data

We utilized two large multimodal neuroimaging datasets including two imaging modalities: T1-weighted structural MRI (sMRI) and resting-state functional MRI (FMRI). The first dataset is from the UK Biobank study (Miller et al., 2016). 2907 subjects from two sites (age mean ± standard deviation: 62.09 ± 7.32 years; age median: 63 years; age range: 46 – 79 years; 1452 males, 1455 females) were used for formal analysis after excluding subjects with more than 4% missing phenotype measures (Smith et al., 2015). The second dataset includes 999 patients and controls (age mean ± standard deviation: 38.61 ± 13.13 years; age median: 39 years; age range: 15 – 65 years; 625 males, 374 females; 538 controls, 337 patients diagnosed with schizophrenia, 63 patients with bipolar disorder, 11 patients with schizoaffective disorder, 28 schizoaffective bipolar-type probands, and 22 schizoaffective depression-type probands) combined across several studies, including Bipolar and Schizophrenia Network for Intermediate Phenotypes (BSNIP) (Tamminga et al., 2014), Center for Biomedical Research Excellence (COBRE) (Aine et al., 2017), Function Biomedical Informatics Research Network (FBIRN) (Keator et al., 2016), and Maryland Psychiatric Research Center (MPRC). For each dataset, we preprocessed sMRI and fMRI to obtain the gray matter (GM) and mean-scaled amplitude of low frequency fluctuations (mALFF) feature maps, respectively. We resampled each GM or mALFF feature map to 3 × 3 × 3mm^3^ resolution and applied a group-level GM mask on the feature map, resulting in 44318 voxels. Data acquisition and preprocessing details are described in Appendix A.

Next, for each data modality in each dataset, we performed variance normalization (removed mean and divided by standard deviation) for each subject, and then removed the mean across all subjects for each voxel. Lastly, we regressed out site effects for each dataset as follows:

(6) $$X^{\left[m\right]}\leftarrow X^{\left[m\right]}-X^{\left[m\right]}L{\left(L^{⊤}L\right)}^{-1}L^{⊤},$$

where L=[1,ℓ], with $1\in R^{N}$ being a column vector of ones and l being one-hot encoded site labels.

### 2.3 Experiments

#### 2.3.1 Synthetic data experiment

We first verified whether the proposed approaches including MSIVA can identify and distinguish the correct subspace structure (i.e. the one used to generate the data) from the incorrect ones in synthetic data. For each of the five subspace structures $\left(S_{1}-S_{5}\right)$ described in Section 2.1.1, we generated a synthetic dataset where the data distribution is defined by the corresponding subspace structure. Next, we conducted experiments on all combinations of five subspace structures (Figure 1) and three initialization workflows (Figure 2). Finally, we visualized the interference matrices ${\overset{ˆ}{W}}^{\left[m\right]}A^{\left[m\right]}$^2^ to confirm if the subspace structures were recovered. We quantitatively measured the normalized multidataset Moreau-Amari intersymbol interference (ISI) (Amari et al., 1996; Macchi & Moreau, 1995; Silva et al., 2020), a metric to evaluate the residual interference between the estimated sources and the ground-truth sources:

(7) $$\text{ISI}\left(H\right)=\frac{1}{2K\left(K-1\right)}\left[\sum_{i=1}^{K}\left(-1+\sum_{j=1}^{K}\frac{\left|h_{ij}\right|}{\underset{k}{\text{max}}\left|h_{ik}\right|}\right)+\sum_{j=1}^{K}\left(-1+\sum_{i=1}^{K}\frac{\left|h_{ij}\right|}{\underset{k}{\text{max}}\left|h_{kj}\right|}\right)\right],$$

where H is a matrix with elements $h_{ij}=1^{⊤}\left|P_{i}\overset{ˆ}{W}AP_{j}\right|1$, the sum of of absolute values from all elements corresponding to subspaces i and j in the interference matrix $\overset{ˆ}{W}A$.

We also reported the corresponding MISA loss value defined in Equation 5. When evaluating method performance on synthetic data, we prioritize the ISI metric and interference matrix as they leverage the ground-truth information, and examine if the loss value is consistent with these metrics.

#### 2.3.2 Neuroimaging data experiment

We performed experiments on each of two multimodal neuroimaging datasets separately, using each of the same five candidate subspace structures $S_{1}-S_{5}$, and identified the optimal subspace structure as the one yielding the lowest final MISA loss value. Note that the ISI is unavailable because the ground-truth subspace structure is unknown in real data.

In addition, to evaluate cross-modal subspace alignment, we computed cross-modal source correlation using both the *linear* Pearson correlation coefficient and the *nonlinear* randomized dependence coefficient (RDC) (Lopez-Paz et al., 2013). Next, we calculated the mean correlation coefficient (MCC) summary for each subspace structure in a two-stage manner: we first calculated the aggregated correlation in each cross-modal subspace, and then computed the final MCC as the mean of the aggregated correlations across all cross-modal subspaces. This two-stage estimation ensures a balanced contribution from subspaces of different dimensions. Let the cross-modal correlation in the kth cross-modal subspace be $R_{k}\in R^{C_{k}\times C_{k}}$, then

(8) $$\text{MCC}\left(R\right)=\frac{1}{K}\overset{K}{\underset{k=1}{\sum }}\frac{1}{2d_{k}}\overset{C_{k}}{\underset{i=1}{\sum }}\left(\text{max}\left(R_{k\left[i,:\right]}\right)+\text{max}\left(R_{k\left[:,i\right]}\right)\right),$$

where $C_{k}$ is the number of sources in the kth subspace, $d_{k}$ is the dimension of the kth cross-modal subspace, and K is the number of cross-modal subspaces in each subspace structure.

To further assess the cross-modal linkage strength of the estimated subspaces within the optimal subspace structure, separate post-hoc CCA of each cross-modal subspace was used to recover projections with the maximum correlation between the two modalities:

(9) $$\left(p_{k},q_{k}\right)=\underset{p_{k},q_{k}}{\text{arg}\;\text{max}}\;\text{corr}\left(p_{k}^{⊤}{\overset{ˆ}{S}}_{k}^{\left[1\right]},q_{k}^{⊤}{\overset{ˆ}{S}}_{k}^{\left[2\right]}\right),$$

where $p_{k}\in R^{C_{k}}$ and $q_{k}\in R^{C_{k}}$ are the CCA projection vectors for the kth cross-modal subspace, and ${\overset{ˆ}{S}}_{k}^{\left[1\right]}\in R^{C_{k}\times N}$ and ${\overset{ˆ}{S}}_{k}^{\left[2\right]}\in R^{C_{k}\times N}$ are the recovered sources in the kth cross-modal subspace for two modalities. After estimation, post-CCA sources in the kth cross-modal subspace are obtained as $p_{k}^{⊤}{\overset{ˆ}{S}}_{k}^{\left[1\right]}$ and $q_{k}^{⊤}{\overset{ˆ}{S}}_{k}^{\left[2\right]}$. This assessment is sensible because linear transformations of individual sources within the same subspace are considered equivalently optimal^3^ (Cardoso, 1998; Szabó et al., 2012).

### 2.4 Brain-phenotype prediction

To evaluate the association between phenotype measures and cross-modal post-CCA sources, we performed age prediction and sex classification tasks for the UKB dataset, as well as age prediction and binary diagnosis classification tasks (controls vs patients with SZ) for the patient dataset. Specifically, we trained a ridge regression model to predict age and a support vector machine with a linear kernel to classify sex groups or diagnosis groups. For the UKB dataset, 2907 subjects were stratified into a training set of 2000 subjects and a holdout test set of 907 subjects. For the patient dataset, 999 subjects were stratified into a training set of 699 subjects and a holdout test set of 300 subjects in the age prediction task; 875 controls and SZ patients were grouped into a training set of 612 subjects and a test set of 263 subjects in the diagnosis classification task. We performed 10-fold cross-validation to choose the best hyperparameter (regularization parameter set: {0.1,0.2, …, 1}) on the training set, then trained the model using all training subjects and evaluated it on the holdout test set. Age regression performance was measured by mean absolute error (MAE) between predicted age and chronological age. Sex or diagnosis classification performance was assessed via *balanced* accuracy, i.e. 0.5×(true positive rate + true negative rate).

### 2.5 Brain-age delta analysis on UK Biobank data

A key benefit of MSIVA is that the estimated multimodal sources are more expressive by leveraging higher-dimensional (≥2D) cross-modal subspaces. To demonstrate the utility of higher-dimensional subspaces, we proposed to conduct a two-stage *voxelwise* brain-age delta analysis using the UKB estimated sources from the optimal subspace structure. For each voxel in the reconstructed subspace (${\overset{ˆ}{X}}_{k}^{\left[m\right]}={\overset{ˆ}{A}}_{k}^{\left[m\right]}{\overset{ˆ}{S}}_{k}^{\left[m\right]}$^4^), we estimated an initial age delta at the first stage and corrected it for age dependence and other confound variables at the second stage (Smith et al., 2019, 2020):

(10) $$\delta_{1}={\hat{X}}_{i}\beta_{1}-y,$$

(11) $$\delta_{2}=\delta_{1}-Y\beta_{2},$$

where ${\overset{ˆ}{X}}_{i}$ indicates the i-th voxel’s reconstructed patterns from each subspace. Namely, they include SVD-shared^5^ patterns from each cross-modal subspace, reconstructed sMRI patterns from each cross-modal subspace, and reconstructed data from each unimodal subspace (see Appendix B for more details). $y\in R^{N}$ is the demeaned chronological age. $Y\in R^{N\times 10}$ includes the confound variables: the demeaned linear, quadratic, cubic age terms, sex, the interaction between sex and each of the three age terms, the framewise displacement variable, and the spatial normalization variables from sMRI and fMRI. An advantage of the procedure described in Smith et al., 2019, 2020 is that it yields a breakdown of $\delta_{2}$ per predictor in ${\overset{ˆ}{X}}_{i}$. Lastly, we partialized $\delta_{2}$ to remove residual associations between each predictor and the other predictors, obtaining the partialized brain-age delta, $\delta_{2p}$.

We then correlated the voxelwise brain-age delta $\delta_{2p}$ with 25 non-imaging phenotype variables such as lifestyle factors and cognitive test scores (see Appendix C for the full list of phenotype variables) to investigate multimodal brain-phenotype relationships. This voxelwise brain-age delta analysis allows us to visualize a voxel-level spatial map showing how each phenotype variable relates to the difference between chronological and estimated brain age.

## 3 Results

### 3.1 MSIVA identifies the ground-truth subspace structure in synthetic data

**Figure 3:**
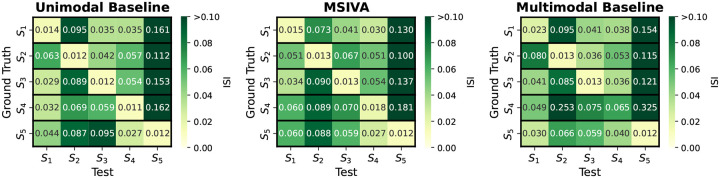
Synthetic data: ISI (lower is better). Each row represents the ground-truth subspace structure used to generate the data and each column represents the test subspace structure used to fit the model. If a workflow could correctly identify all ground-truth subspace structures, the lowest ISI values would align along the main diagonal. The unimodal initialization workflow (PCA+ICA) and the MSIVA initialization workflow (MGPCA+ICA) led to the lowest ISI values (≤ 0.02) along the main diagonal, indicating that these two approaches successfully identified the correct ground-truth subspace structures from the incorrect ones. However, the multimodal initialization workflow (MGPCA+GICA) failed to detect the subspace structure $S_{4}$ with a high ISI value (0.065) in the main diagonal. Thus, MSIVA and the unimodal baseline are considered better than the multimodal baseline.

We first verified whether the proposed approaches, including MSIVA and baseline methods, can identify the correct subspace structures used for data generation in synthetic datasets. As shown in Figure 3, the unimodal initialization workflow (PCA+ICA) and the MSIVA initialization workflow (MGPCA+ICA) led to the lowest ISI values (≤ 0.02) along the main diagonal, demonstrating that both approaches can correctly recover the ground-truth subspaces when the correct subspace structure is provided. The multimodal initialization workflow, on the other hand, showed suboptimal performance with an elevated ISI value (0.065) along the main diagonal and was thus excluded from subsequent neuroimaging data experiments. According to Table 1, the loss values are largely consistent with the ISI results, except that the loss value incorrectly implies that MSIVA $S_{5}$ is a better fit when $S_{4}$ is used to generate the data. The loss values obtained with the multimodal initialization workflow (MGPCA+GICA) failed to detect the ground-truth subspace structures containing 4D subspace(s), i.e. $S_{1}$ and $S_{4}$.

**Figure 4:**
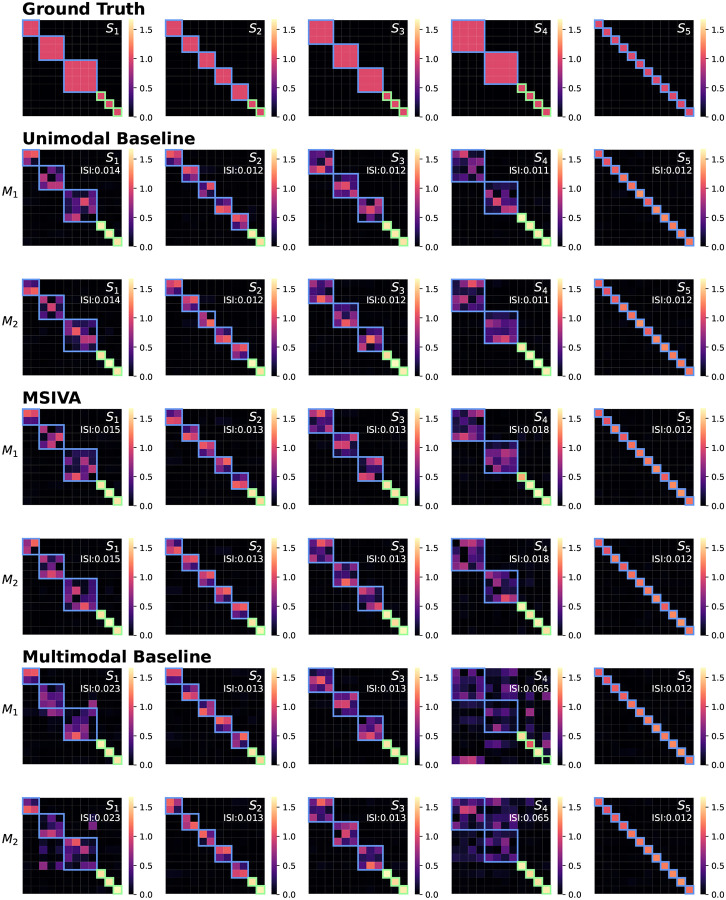
Synthetic data: Interference matrices ${\hat{\text{W}}}^{\left[m\right]}{\text{A}}^{\left[m\right]}$ corresponding to the diagonal ISI values in Figure 3. Cross-modal subspaces are highlighted in blue while unimodal subspaces are highlighted in green. The same subspace permutation was applied for both modalities for ease of interpretation. The correct subspace structures were identified and aligned across both modalities by three workflows (rows II-VII), in accordance with the ground-truth simulation design (row I), except that the multimodal baseline failed to estimate $S_{1}$ and $S_{4}$ (rows VI-VII).

As presented in Figure 4, the recovered subspace structures from MSIVA (rows IV-V) and the unimodal initialization workflow (rows II-III) under the correct subspace structure aligned well with the proposed ground truth (row I), confirming the effectiveness of MSIVA and the unimodal baseline. However, the multimodal initialization workflow (rows VI-VII) could not recover the ground-truth subspace structures for $S_{1}$ and $S_{4}$ even when given the correct subspace structure, indicating that the difficulty of the cross-modal alignment optimization increases in the presence of high-dimensional subspaces.

### 3.2 MSIVA better detects the latent subspace structure in neuroimaging data

**Figure 5:**
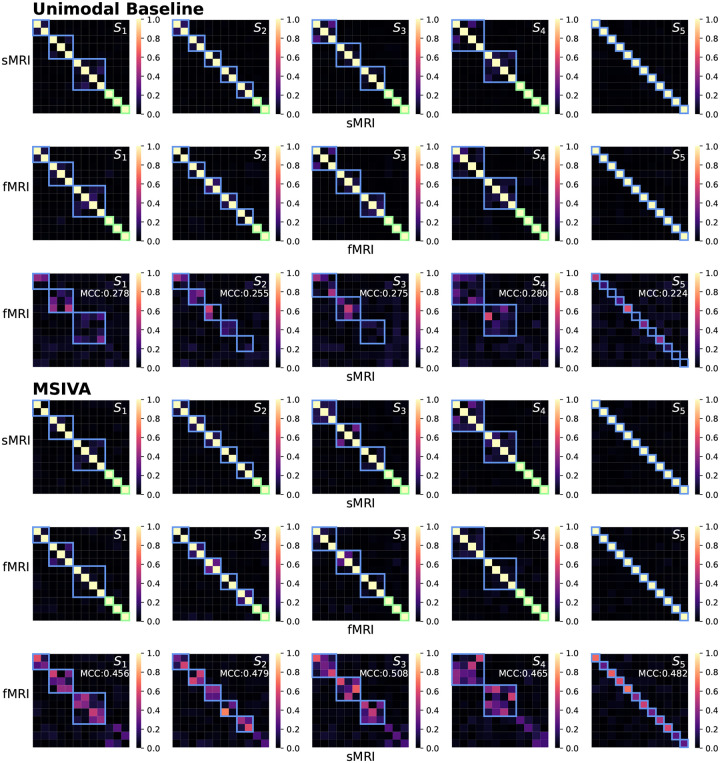
UKB neuroimaging data: Within-modal Pearson correlations (rows I-II and IV-V) and cross-modal Pearson correlations (rows III and VI) of the recovered sources before applying post-hoc CCA. Cross-modal subspaces are highlighted in blue while unimodal subspaces are highlighted in green. Within-modal self-correlation patterns indicate negligible residual dependence between subspaces (rows I-II and IV-V). MSIVA shows stronger cross-modal correlations (higher MCCs) than the unimodal baseline for all predefined subspace structures (row VI vs row III).

We next applied MSIVA and the unimodal baseline on two large multimodal neuroimaging datasets separately – the UK Biobank (UKB) dataset and the combined schizophrenia (SZ) dataset – to detect their latent subspace structures. In the UKB neuroimaging dataset, we observe that within-modal self-correlation patterns (Figure 5, rows I-II and IV-V) indicate negligible residual dependence between subspaces, as expected (dependence within subspaces is acceptable, but not between them). We note that MSIVA recovered stronger cross-modal correlations (higher MCCs) than the unimodal baseline for all predefined subspace structures (Figure 5, row VI vs row III). Results from the nonlinear dependence measure also confirm that sources in cross-modal subspaces are linked across modalities, while sources in different subspaces within each modality are independent (Appendix D Figure 14). Among all combinations of two initialization workflows and five candidate subspace structures, MSIVA with the subspace structure $S_{2}$ outputs the lowest final MISA loss value 46.775 (Table 2), suggesting that MSIVA $S_{2}$ best fits the latent structure of this dataset.

**Figure 6:**
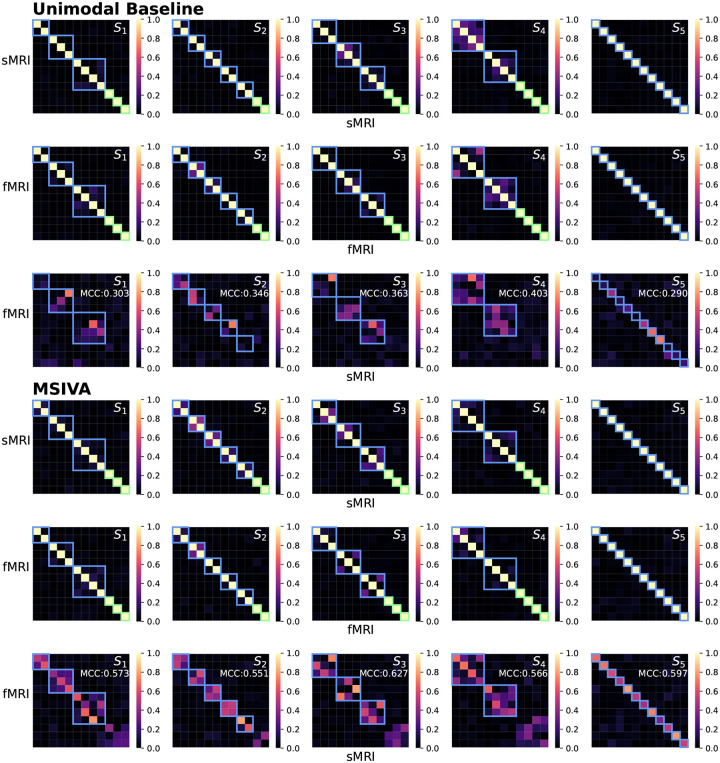
Patient neuroimaging data: Within-modal Pearson correlations (rows I-II and IV-V) and cross-modal Pearson correlations (rows III and VI) of the recovered sources before applying post-hoc CCA. Cross-modal subspaces are highlighted in blue while unimodal subspaces are highlighted in green. Within-modal self-correlation patterns indicate negligible residual dependence between subspaces (rows I-II and IV-V). MSIVA shows stronger cross-modal correlations (higher MCCs) than the unimodal baseline for all predefined subspace structures (row VI vs row III).

Similarly, in the patient dataset, MSIVA shows stronger cross-modal correlations (dependence) for all five subspace structures (Figures 6 and 15, row VI vs row III). Same as the UKB dataset, MSIVA $S_{2}$ yields the lowest final loss value 45.674 in all cases (Table 2). In addition, relative to the loss values in Table 1, the MSIVA loss values are consistently lower than the unimodal ones. These results imply that MSIVA and the subspace structure $S_{2}$ with five linked 2D subspaces can better fit the statistical relationships in these two multimodal neuroimaging datasets.

### 3.3 MSIVA reveals linked phenotypic and neuropsychiatric biomarkers

**Figure 7:**
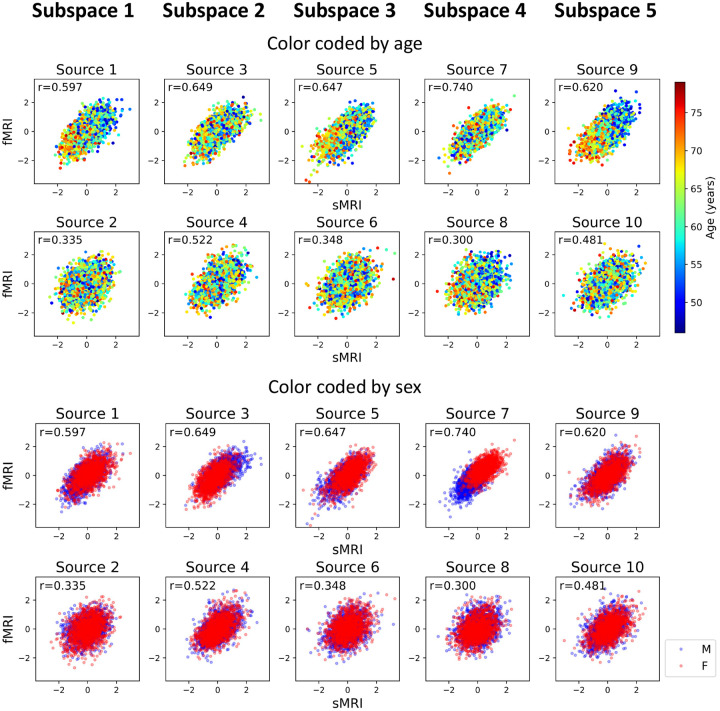
UKB neuroimaging data: Post-CCA sources from MSIVA $S_{2}$ cross-modal subspaces, color coded by age and sex. Rows I and II show the age effect, while rows III and IV show the sex effect. In particular, subspaces 1, 3, 4 and 5 are associated with aging (especially cross-modal source 9 in subspace 5), while subspaces 2 and 4 show the sex difference (especially cross-modal source 7 in subspace 4).

**Figure 8:**
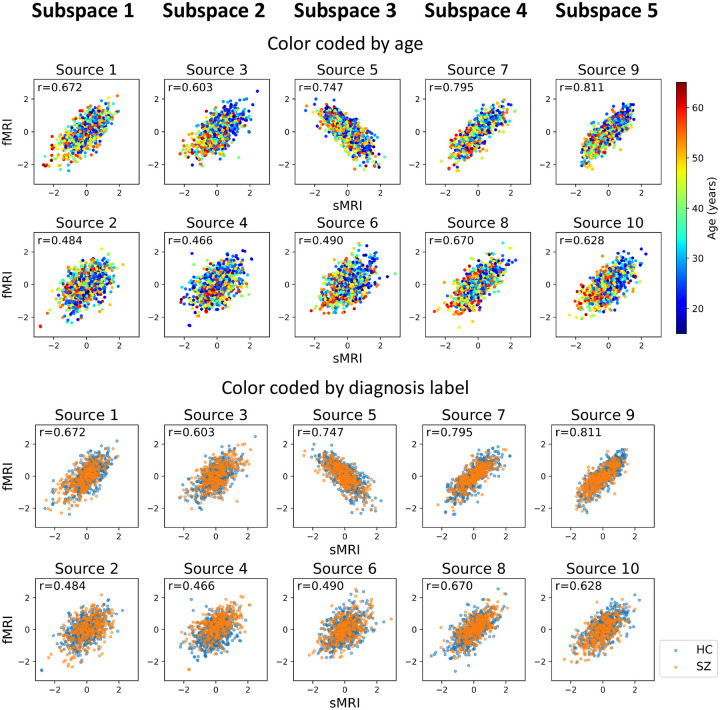
Patient neuroimaging data: Post-CCA sources from MSIVA $S_{2}$ cross-modal subspaces, color coded by age and diagnosis labels. Rows I and II show the age effect, while rows III and IV show the SZ effect. In particular, subspaces 2 and 5 are associated with the age- (especially cross-modal source 3 in subspace 2 and sources 9 and 10 in subspace 5) and SZ-related effects (especially cross-modal source 4 in subspace 2 and sources in subspace 5).

After identifying the neuroimaging sources, we asked whether the linked subspaces are biologically meaningful. To answer this question, we evaluated the brain-phenotype relationships between phenotype variables and neuroimaging sources estimated by MSIVA (with the optimal subspace structure $S_{2}$ selected based on Table 2). In the UKB dataset, visual inspection of individual variability from the cross-modal CCA projections in each linked subspace (Figure 7) suggests that subspaces 1, 3, 4 and 5 are associated with aging (especially cross-modal source 9 in subspace 5), while subspaces 2 and 4 show the sex effect (especially cross-modal source 7 in subspace 4). Furthermore, we used the post-CCA sources from each linked subspace to predict age and sex. The age regression and sex classification performance also confirmed that subspace 5 is strongly associated with age while subspace 2 is strongly associated with sex (Table 3). More specifically, the age prediction MAE in subspace 5 is the lowest (5.378 years), and the sex classification balanced accuracy is the highest in subspace 4 (79.933%). As for the patient dataset, according to the cross-modal CCA projections in each linked subspace (Figure 8), we observe the age effect in source 3 from subspace 2, and both sources 9 and 10 from subspace 5. We also find the SZ-related effect in source 4 from subspace 2, as well as sources 9 and 10 from subspace 5. These associations were verified by the age regression and diagnosis classification results (Table 3).

**Figure 9:**
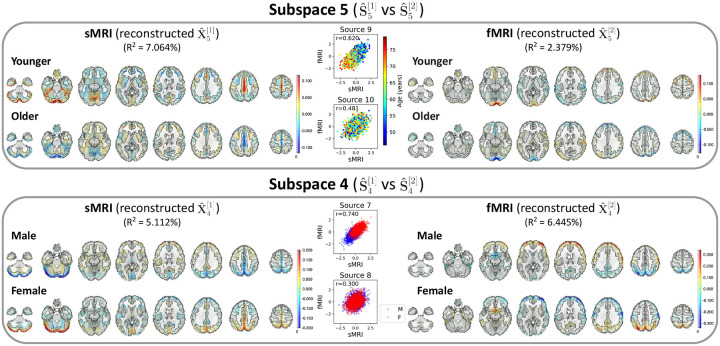
UKB neuroimaging data: Spatial maps of group-specific reconstructed data from MSIVA $S_{2}$ sources related to age and sex effects. Axial slices show the geometric median of the reconstructed data (${\hat{\text{X}}}_{k}^{\left[m\right]}$) for each modality (sMRI or fMRI) and each group (younger: 46 – 63 years, older: 63 – 79 years; male or female). Voxel intensity is mapped to both color hue and opacity. The contours highlight the brain areas where voxelwise cross-modal correlations are significant for each group (P<0.01, Bonferroni correction for 44318 voxels). Scatter plots show post-CCA sources color-coded by age or sex. The reported $R^{2}$ indicates the proportion of variance captured by the subspace in each modality.

**Figure 10:**
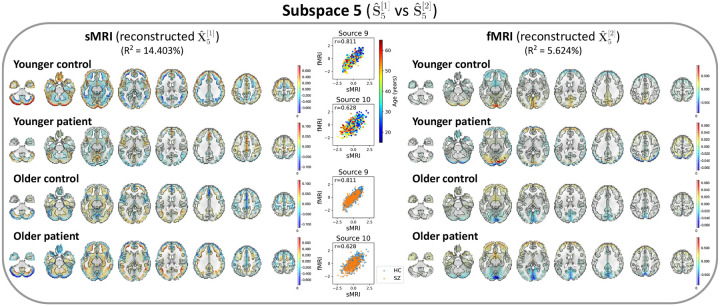
Patient neuroimaging data: Spatial maps of group-specific reconstructed data from MSIVA $S_{2}$ sources related to age and SZ interaction effects. Axial slices show the geometric median of the reconstructed data (${\overset{ˆ}{X}}_{k}^{\left[m\right]}$) for each modality (sMRI or fMRI) and each group (younger: 15 – 39 years, older: 39 – 65 years; control or patient). Voxel intensity is mapped to both color hue and opacity. The contours highlight the brain areas where voxelwise cross-modal correlations are significant for each group (P<0.01, Bonferroni correction for 44318 voxels). Scatter plots show post-CCA sources color-coded by age or diagnosis label. The reported $R^{2}$ indicates the proportion of variance captured by the subspace in each modality.

Next, we utilized a dual-coded visualization (Allen et al., 2012) for the modality- and group-specific geometric median spatial maps of the reconstructed data ${\overset{ˆ}{X}}_{k}^{\left[m\right]}={\overset{ˆ}{A}}_{k}^{\left[m\right]}{\overset{ˆ}{S}}_{k}^{\left[m\right]}$ from each representative subspace k (Figures 9 and 10). Voxel intensity is mapped to both color hue and opacity. The contours highlight brain regions where voxelwise cross-modal correlations are significant for each linked subspace and each group (P<0.01, Bonferroni correction for 44318 voxels), after eliminating small clusters of voxels by applying morphological dilation and erosion to the original contours.

In the UKB dataset, source 9 from subspace 5 shows the strongest age effect, while source 7 from subspace 4 shows the strongest sex effect (Figure 9). *Subspace 5*: We observe age effects in the cerebellum, precentral gyrus, cingulate gyrus, and paracingulate gyrus in sMRI; the occipital pole, lateral occipital cortex, superior frontal gyrus, and precuneus in fMRI. In particular, younger subjects (whose age is less than the median age in the UKB dataset, i.e. 46 – 63 years) show higher positive voxel intensities in these areas, while older subjects (whose age is greater than or equal to the median age in the UKB dataset, i.e. 63 – 79 years) show negative intensities in the same areas. Several brain regions identified in our study align with previous findings. For example, cerebellar volume has been reported to be associated with age-related decline (Jernigan et al., 2001; Luft et al., 1999; Romero et al., 2021). Hogstrom et al., 2013 has observed strong age effect in the precentral gyrus and weak age effect in the cingulate gyrus from structural brain imaging. Also, functional network research has identified significant association with aging in the occipital lobe (Scheinost et al., 2015). *Subspace 4*: Sex effects can be seen in the frontal lobe, occipital lobe, and precuneus in both sMRI and fMRI. Female participants have strong positive intensities in the cerebellum (sMRI), lateral occipital cortex (fMRI), subcallosal area (fMRI), and precuneus cortex (sMRI and fMRI), and negative intensities in the frontal pole and postcentral gyrus (fMRI). We observe the opposite patterns in male participants. Previous studies have also found sex differences in the gray matter volume of the cerebellum (Fan et al., 2010) and the precuneus cortex (Ruigrok et al., 2014), as well as in the frontal and occipital areas via functional measures (Tian et al., 2011). Spatial maps for the other MSIVA $S_{2}$ cross-modal subspaces in the UKB dataset are presented in Appendix E Figure 16.

In the patient dataset, sources from subspace 5 are significantly associated with different age and diagnosis groups (Figure 10). The younger control participants show high positive intensities in the cerebellum, temporal pole, and frontal operculum cortex in sMRI; the lingual gyrus, occipital pole, and precuneus cortex in fMRI. They also exhibit negative intensities in the middle temporal gyrus, inferior temporal gyrus, and occipital fusiform gyrus in sMRI. Additionally, we observe both strong positive and negative voxel intensities in the frontal lobe of sMRI. The younger patients show slightly positive intensities in the cerebellum, paracingulate gyrus, insular cortex, supplementary motor cortex, and cingulate gyrus in sMRI, and the occipital fusiform gyrus in fMRI, but show negative intensities in the lateral occipital cortex and occipital pole in fMRI. The older group (whose age is greater than or equal to the median age in the patient dataset, i.e. 39 – 65 years) has decreased intensities in the cerebellum, paracingulate gyrus, insular cortex in sMRI, as well as in the lingual gyrus, precuneus cortex, and occipital pole in fMRI. In particular, we observe reduced sMRI intensities in the cerebellum of the patient group compared to their age-matched control group. This result aligns with the previous finding that the cerebellar gray matter volume is significantly reduced in SZ patients (Moberget et al., 2018; Picard et al., 2008). We also note that younger patients with SZ show negative fMRI intensities in the lateral occipital cortex and occipital pole compared to younger controls, and the intensities in these areas are further reduced in older patients. This finding may be explained by previous research that SZ is associated with impaired function of the visual pathway (Martínez et al., 2008). Spatial maps for the other MSIVA $S_{2}$ linked subspaces in the patient dataset are shown in Appendix E Figure 17.

In addition, we note that the number of voxels with significant cross-modal correlations (P<0.01, Bonferroni correction for 44318 voxels) for older patients diagnosed with SZ (25623) is 18.6% less than their age-matched control subjects (31482) in subspace 5. Particularly, the brain areas with reduced structure-function agreement include the insular cortex, lingual gyrus, occipital pole, inferior frontal gyrus, and paracingulate gyrus. Apart from subspace 5, we observe consistent reductions in the number of voxels with significant cross-modal correlations for older patients with SZ in the other three linked subspaces (Appendix E Figure 18 subspaces 1–3), suggesting decreased coupling between brain structure and function for older patients.

### 3.4 Brain-age gap is associated with lifestyle factors and cognitive functions

**Figure 11:**
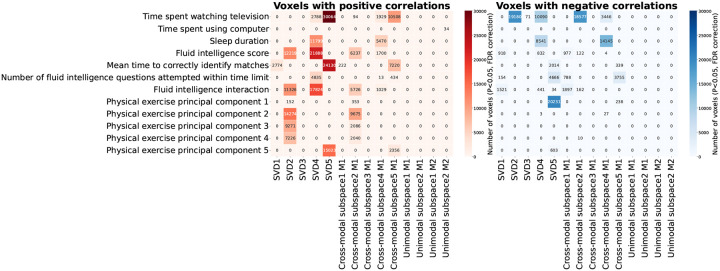
Number of voxels with significant Pearson correlation between corrected brain-age delta $\delta_{2p}$ and phenotype variables. Brain-age gap shows significant positive (left) and negative (right) associations with phenotype variables including physical exercise, time spent watching TV, sleep duration, and fluid intelligence (P<0.05, false discovery rate correction for 44318 voxels, 25 phenotype variables, and 14 predictors).

We performed a two-stage voxelwise brain-age delta analysis using the UKB sources estimated by MSIVA using the optimal subspace structure $S_{2}$ (see Appendix B for details). We investigated whether the brain-age gap shows association with other phenotype variables by measuring Pearson correlation between $\delta_{2p}$ and each phenotype variable for each voxel. To examine effects specific to shared multimodal variability, we applied voxelwise singular value decomposition (SVD) to the combined reconstructed data from both modalities (${\overset{ˆ}{X}}_{k}^{\left[1\right]}$ and ${\overset{ˆ}{X}}_{k}^{\left[2\right]}$) for each of the five cross-modal subspaces. We find that the brain-age deltas corresponding to the top SVD-shared voxel-level features from cross-modal subspaces 2, 4, 5 are significantly associated with various phenotype variables, including time spent watching TV, sleep duration, fluid intelligence, and physical exercise (Figure 11). In particular, predictor 5 (SVD-shared feature from cross-modal subspace 5), which shows the strongest age association (Table 3 and Appendix B Figure 13), positively correlates with time spent watching TV and mean time to correctly identify matches (cognitive performance), and negatively correlates with the first principal component of physical exercise variables.

**Figure 12:**
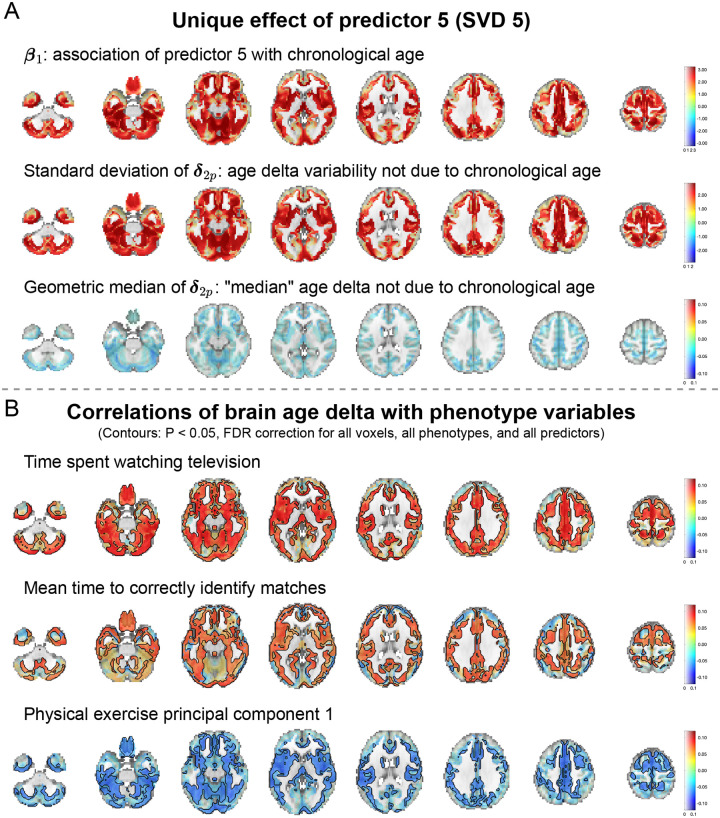
Spatial maps of predictor 5 (SVD 5) from brain-age delta analysis. (A) Spatial maps of $\beta_{1}$, standard deviation of $\delta_{2p}$, and geometric median of $\delta_{2p}$. Voxel value is mapped to both color hue and opacity. (B) Voxelwise correlations between $\delta_{2p}$ and phenotype variables time spent watching TV, mean time to correctly identify matches, the first principal component of physical exercise variables. The voxelwise correlation is mapped to both color hue and opacity. The contours outline the brain regions where the correlations are significant (P<0.05, false discovery rate correction for 44318 voxels, 25 phenotype variables, and 14 predictors). 14431 voxels overlap within the contours in these three spatial maps.

**Figure 13:**
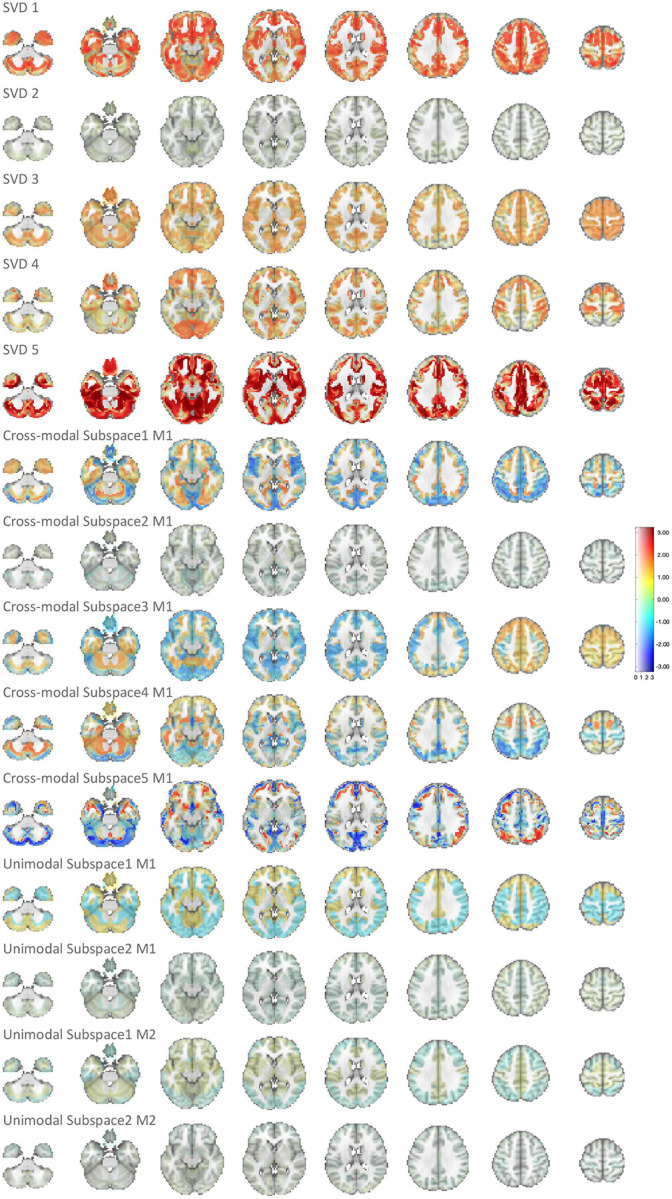
Spatial maps of $\beta_{1}$ in voxelwise brain-age delta analysis. Voxel intensity is mapped to both color hue and opacity. SVD 5 shows the strongest age association among all predictors.

**Figure 14:**
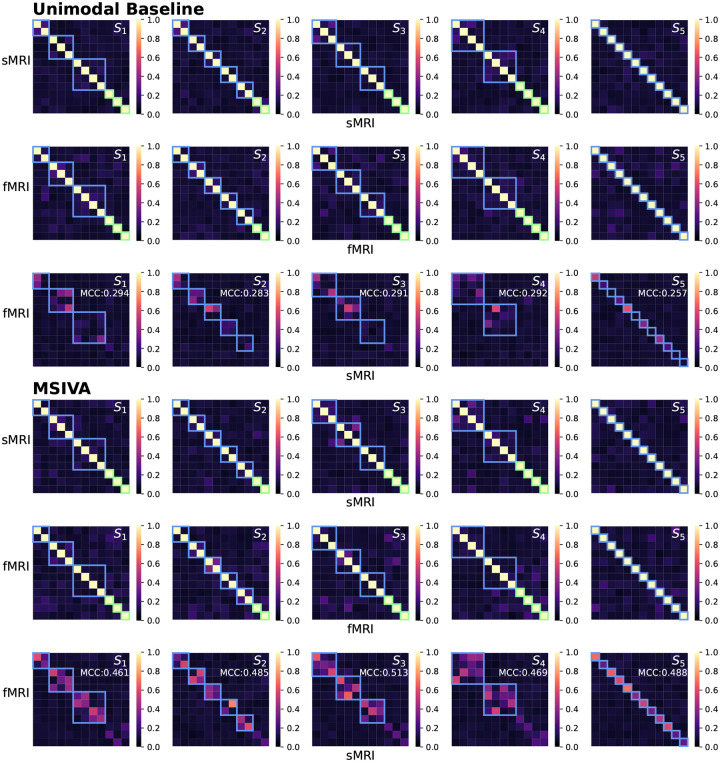
UKB neuroimaging data: Within-modal RDCs (rows I-II and IV-V) and cross-modal RDCs (rows III and VI) of the recovered sources before applying post-hoc CCA. Cross-modal subspaces are highlighted in blue while unimodal subspaces are highlighted in green. Within-modal self-correlation patterns show very weak residual dependence between subspaces (rows I-II and IV-V). MSIVA exhibits stronger cross-modal correlations than the unimodal baseline for all predefined subspace structures (row VI vs row III).

**Figure 15:**
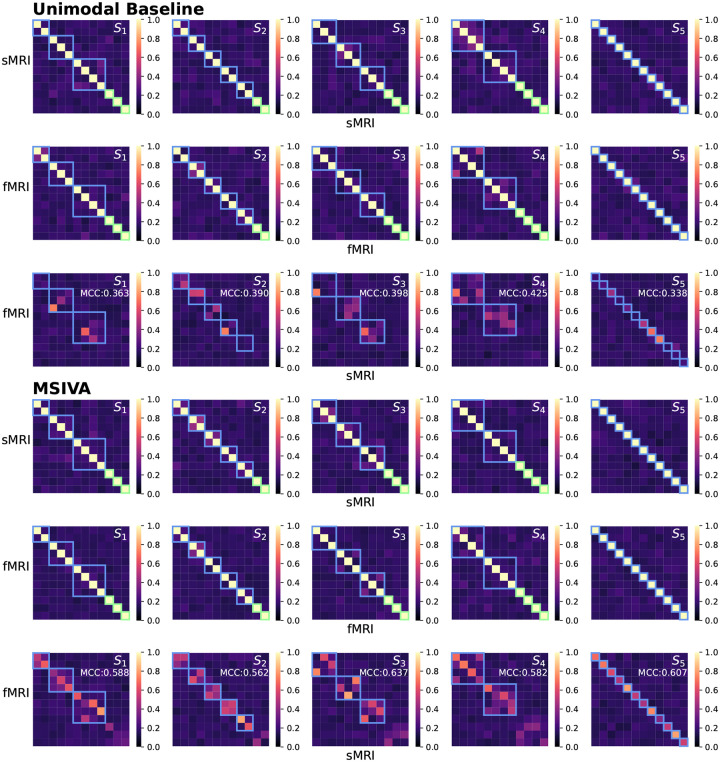
Patient neuroimaging data: Within-modal RDCs (rows I-II and IV-V) and cross-modal RDCs (rows III and VI) of the recovered sources before applying post-hoc CCA. Cross-modal subspaces are highlighted in blue while unimodal subspaces are highlighted in green. Within-modal self-correlation patterns show weak residual dependence between subspaces (rows I-II and IV-V). MSIVA shows stronger cross-modal correlations than the unimodal baseline for all predefined subspace structures (row VI vs row III).

**Figure 16:**
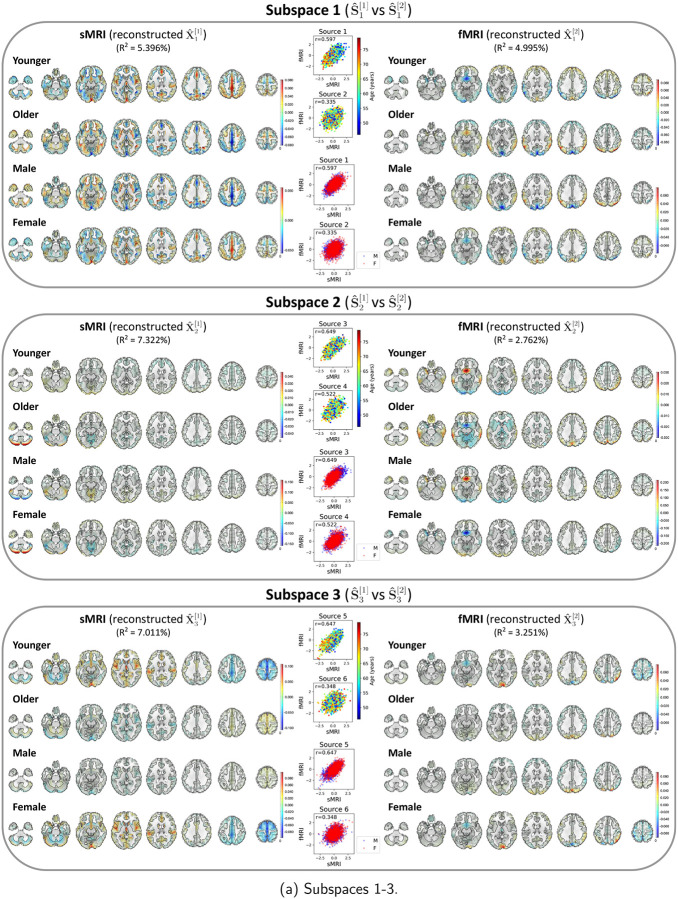

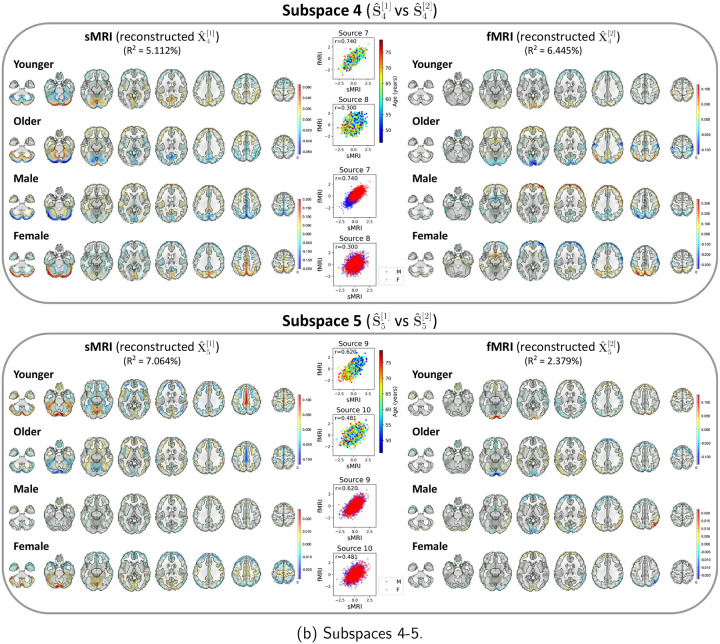
UKB neuroimaging data: Spatial maps of group-specific reconstructed data from MSIVA $S_{2}$ sources related to age and sex effects. Axial slices show the geometric median of the reconstructed data (${\hat{\text{X}}}_{k}^{\left[m\right]}$) for each modality (sMRI or fMRI) and each group (younger: 46 – 63 years, older: 63 – 79 years; male or female). Voxel intensity is mapped to both color hue and opacity. The contours highlight the brain areas where voxelwise cross-modal correlations are significant for each group (P<0.01, Bonferroni correction for 44318 voxels). Scatter plots show post-CCA sources color-coded by age or sex. The reported $R^{2}$ indicates the proportion of variance captured by the subspace in each modality.

**Figure 17:**
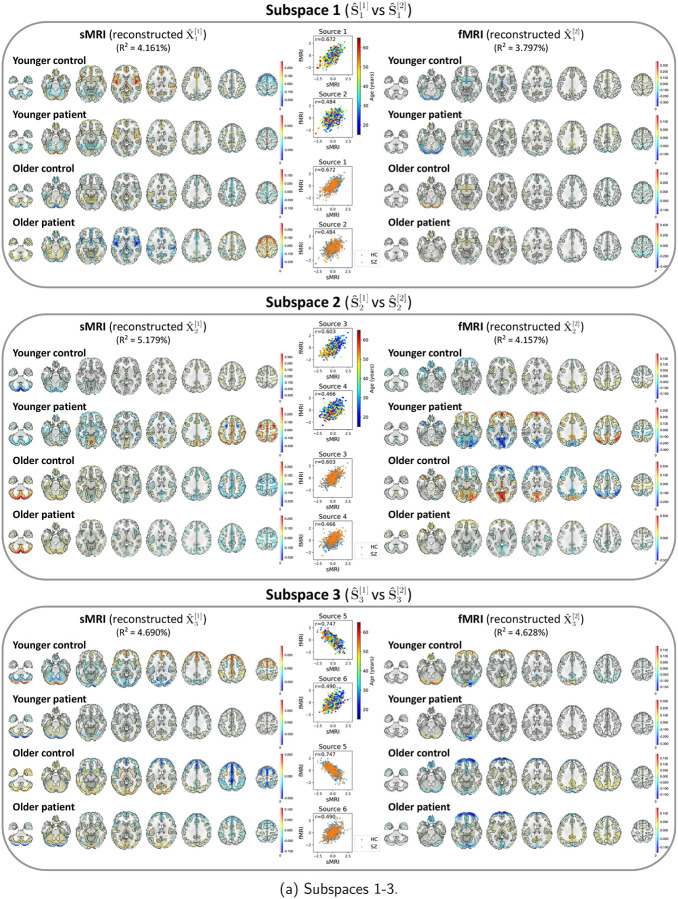

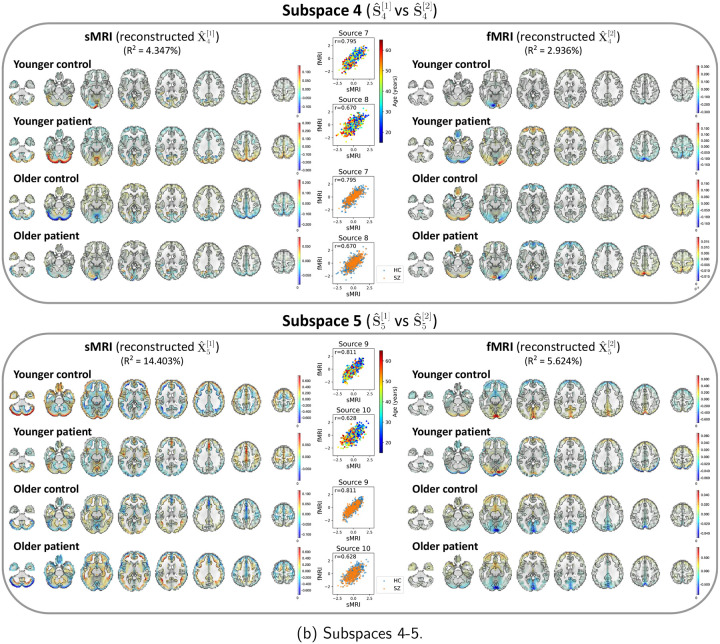
Patient neuroimaging data: Spatial maps of group-specific reconstructed data from MSIVA $S_{2}$ sources related to age and SZ interaction effects. Axial slices show the geometric median of the reconstructed data (${\overset{ˆ}{X}}_{k}^{\left[m\right]}$) for each modality (sMRI or fMRI) and each group (younger: 15 – 39 years, older: 39 – 65 years; control or patient). Voxel intensity is mapped to both color hue and opacity. The contours highlight the brain areas where voxelwise cross-modal correlations are significant for each group (P<0.01, Bonferroni correction for 44318 voxels). Scatter plots show post-CCA sources color-coded by age or diagnosis label. The reported $R^{2}$ indicates the proportion of variance captured by the subspace in each modality.

**Figure 18:**
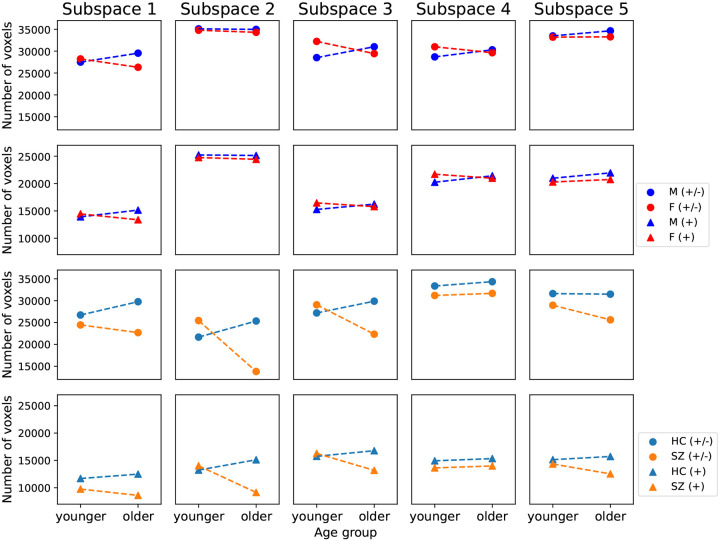
Number of voxels that show significant cross-modal correlations for age and sex groups in the UKB dataset (rows I and II), and for age and diagnosis groups in the patient dataset (rows III and IV). Rows I and III display the number of voxels with both positive and negative correlations (+/−), while rows II and IV display the number of voxels with only positive correlations (+). The number of voxels for older patients diagnosed with SZ is consistently less than that for their age-matched controls in four of five subspaces, implying reduced brain structure-function coupling in older patients.

We visualize the relevant spatial maps of predictor 5 (SVD 5) in Figure 12. According to Table 3, subspace 5 shows the strongest association with the chronological age. This aligns with the strong $\beta_{1}$ coefficients and $\sigma \left(\delta_{2p}\right)$ spatial maps from the first step of brain-age delta analysis (Figure 12, panel A , rows I and II). The geometric median of brain-age delta $\delta_{2p}$ is slightly negative (Figure 12, panel A, row III), indicating that biological age is slightly lower than chronological age (i.e. the brain appears younger). We also present spatial maps for three phenotype variables that show strong associations with $\delta_{2p}$: time spent watching TV, mean time to correctly identify matches, and the first principal component of physical exercise variables (Figure 12, panel B). Particularly, we observe significant effects in the cerebellum, postcentral gyrus, cingulate gyrus, precuneus cortex, occipital lobe, and caudate nucleus for time to watch TV; the frontal pole, precentral gyrus, and insular cortex for time to identify matches; the cerebellum, occipital fusiform gyrus, and caudate nucleus for physical exercise measure. If the correlation on the spatial map is negative (as in the first principal component of physical exercise), $\delta_{2p}$ decreases as the phenotype score increases and the brain appears younger. If it is positive (as in time to watch TV or identify matches), $\delta_{2p}$ increases as the phenotype score increases and the brain appears older. Therefore, the more physical exercise, the younger the brain looks; the more time spent watching TV or identifying correct matches, the older the brain looks. These findings indicate that increased physical activity and reduced TV time can potentially improve brain health.

## 4 Discussion

We present a novel multivariate methodology, Multimodal Subspace Independent Vector Analysis (MSIVA), to capture both cross-modal and unimodal sources. We first showed that MSIVA successfully identified the ground truth when given the correct subspace structure, according to the ISI and interference matrix results, and verified that the correct subspace structures led to the lowest loss values for all synthetic data experiments, except for one case. We next applied MSIVA to two large multimodal neuroimaging datasets and demonstrated that it better revealed the latent subspace structure, yielding lower loss values compared with the unimodal baseline. Among all combinations of different initialization workflows and subspace structures, MSIVA with the subspace structure $S_{2}$ output the lowest loss value, thus being considered as the best fit to the latent structure in both neuroimaging datasets. The CCA projections within each cross-modal subspace were strongly associated with age, sex and SZ-related effects, as verified through the phenotype prediction tasks. Moreover, the voxelwise brain-age delta analysis on the UKB dataset identified key non-imaging phenotype variables, including lifestyle factors and cognitive performance, that are significantly correlated with voxel-level brain-age gap.

We evaluated three initialization workflows that capture different amounts of joint information. Interestingly, MSIVA outperformed a unimodal baseline and a multimodal baseline. One reason can be that the unimodal baseline uses random initialization without any cross-modal information, leading to potentially unrecoverable misalignment, while the multimodal baseline might overfit the cross-modal information. MSIVA, which captures intermediate level of cross-modal information for initialization, appears to strike the best balance among the three initialization workflows.

Furthermore, MSIVA can be viewed as an extension of MMIVA which 1) uses a different initialization method (MSIVA: MGPCA+ICA initialization; MMIVA: MGPCA+GICA initialization) and 2) allows for arbitrary subspace structures (MSIVA: flexible subspace structures like $S_{1}-S_{5}$ and more; MMIVA: rigid subspace structures like an identity matrix $S_{5}$). To further investigate the relationships between the estimated sources from MSIVA and MMIVA, we compared MSIVA (with the subspace structure $S_{2}$) and MMIVA by using MSIVA $S_{2}$ sources to predict MMIVA sources, as well as using matched MMIVA sources to predict MSIVA $S_{2}$ sources. We find that the pair of MSIVA $S_{2}$ sources from each subspace can predict variability from more than two MMIVA sources, while pairs of matched MMIVA sources can also predict variability from more than two MSIVA $S_{2}$ sources (see Appendix F). Hence, there is no perfect one-to-one mapping between MSIVA $S_{2}$ sources and MMIVA sources. We conclude that MSIVA and MMIVA apportion variability to their sources in different ways. We also note that the mismatch appears to be more pronounced in the patient dataset than in the UKB dataset, which may be related to inherent characteristics of the patient data, such as higher population heterogeneity and smaller sample size.

A limitation of our current work is the subspace structure used in MSIVA. MSIVA selects the best-fitting subspace structure for the data from a predefined set, according to the ISI (when ground-truth is available) or loss value (when ground-truth is *not* available). However, it is not computationally efficient to exhaustively evaluate the merits of other potential subspace structures. Additionally, we make two assumptions on the subspace structure: the cross-modal subspaces have the same dimensionality per modality, and the unimodal subspaces are all one-dimensional. Yet, it is possible that these assumptions might not represent the true underlying structure of the dataset. In future work, we plan to apply data-driven subspace structures such as the NeuroMark template (Du et al., 2020; Fu et al., 2024), or learn the underlying subspace structure from the data directly in an unsupervised manner. In this study, we chose 12 latent sources to approximate each data modality for the sake of computational efficiency during combinatorial optimization, but 12 sources only might not capture the necessary amount of variability in the data to recover all multimodal links (Song et al., 2016). Further workflow optimization is needed to efficiently estimate alignment for subspaces of higher dimensionality.

Although we utilized the loss value to select the optimal subspace structure in neuroimaging data due to the lack of ground-truth information, we notice that the loss value might not always be a gold standard for measuring the goodness of fit. For example, in synthetic data experiments, MSIVA successfully identified $S_{4}$ according to the ISI values (Figure 3) but failed to identify $S_{4}$ according to the loss values (Table 1). Hence, we suggest to comprehensively evaluate method performance using multiple metrics in addition to the loss value, such as the MCC, which measures average cross-modal subspace alignment. Another limitation is the linear mixing assumption in MSIVA. MSIVA assumes that each data modality can be transformed to linearly mixed sources, but the true mixing process in neuroimaging data may be nonlinear, especially considering the multiple nonlinear transformations in FMRI modeling and preprocessing stages. To address this limitation, we are currently working on developing *nonlinear* latent variable models that estimate multimodal sources which are nonlinearly mixed.

## 5 Conclusions

Our proposed multivariate methodology MSIVA effectively captures both within- and cross-modal sources, as well as their underlying subspace structure, from multiple synthetic and neuroimaging datasets. According to brain-phenotype modeling, the estimated sources from the MSIVA cross-modal subspaces are strongly associated with phenotype variables including age, sex, and psychosis. Subsequent brain-age delta analysis shows that voxel-wise brain-age gap in the recovered cross-modal subspaces is related to lifestyle and cognitive function measures. Our results support that MSIVA can be applied to uncover linked phenotypic and neuropsychiatric biomarkers of brain structure and function at the voxel level from multimodal neuroimaging data.

## Figures and Tables

**Figure 19:**
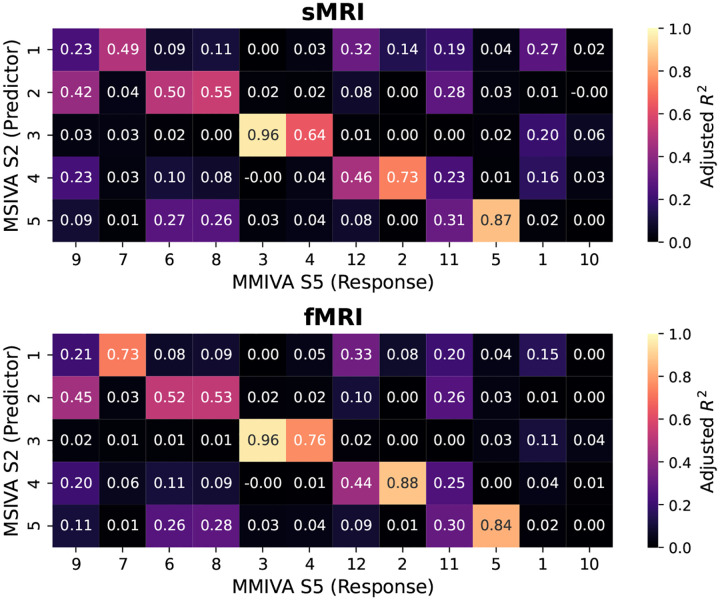
UKB neuroimaging data: Adjusted $R^{2}$ using MSIVA sources to predict MMIVA sources.

**Figure 20:**
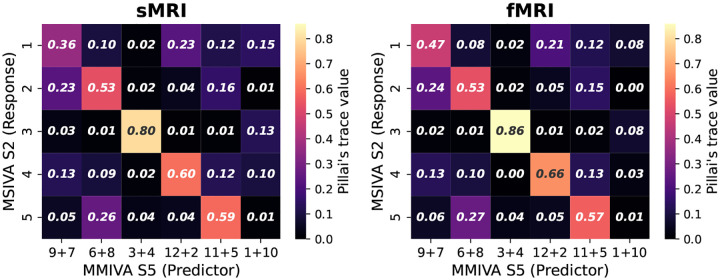
UKB neuroimaging data: Pillai’s trace value using matched MMIVA sources to predict MSIVA sources.

**Figure 21:**
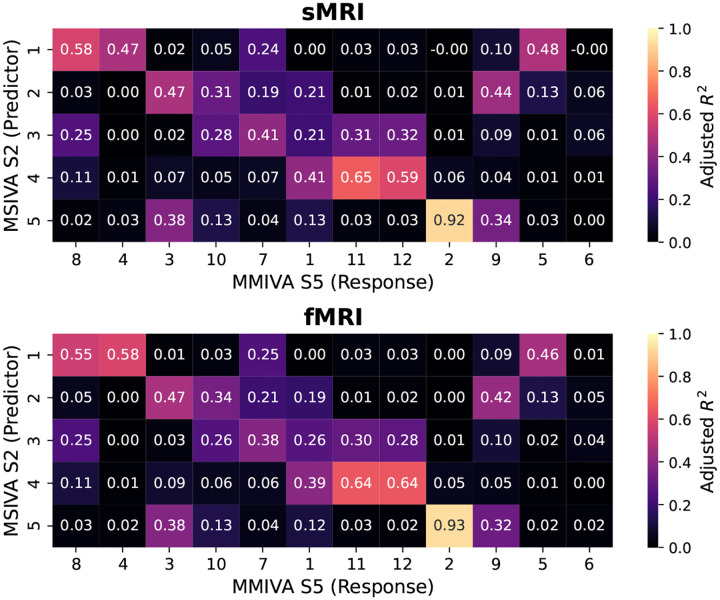
Patient neuroimaging data: Adjusted $R^{2}$ using MSIVA sources to predict MMIVA sources.

**Figure 22:**
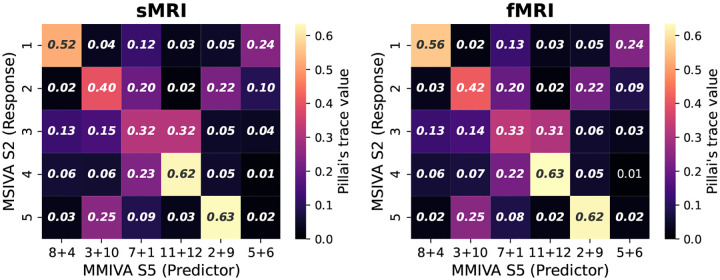
Patient neuroimaging data: Pillai’s trace value using matched MMIVA sources to predict MSIVA sources.

**Table 1:** Synthetic data: Final MISA loss values (lower is better). Each row represents the ground-truth (GT) subspace structure used to generate the data and each column represents the test subspace structure used to fit the model. The lowest loss value along the *row* is highlighted in bold, which determines the selected subspace. Approaches performing consistently well in relation to the ISI in Figure 3 will contain bold loss values *only* along the diagonal. The loss value is largely consistent with the ISI value, except that it incorrectly implies that MSIVA $S_{5}$ is a better fit when $S_{4}$ is used to generate the data. Further, the multimodal baseline results incorrectly imply that $S_{3}$ and $S_{1}$ are better when $S_{1}$ and $S_{4}$ are the ground-truth subspace structures, respectively. Overall, the differences in diagonal loss values between MSIVA and the unimodal baseline appear negligible considering the correspondingly negligible differences in ISI (Figure 3).

Unimodal Baseline	$S_{1}^{\text{Test}}$	$S_{2}^{\text{Test}}$	$S_{3}^{\text{Test}}$	$S_{4}^{\text{Test}}$	$S_{5}^{\text{Test}}$
$S_{1}^{\text{GT}}$	**42.692**	42.884	42.762	42.992	43.230
$S_{2}^{\text{GT}}$	42.649	**42.300**	42.851	42.868	42.918
$S_{3}^{\text{GT}}$	42.720	42.858	**42.635**	43.100	43.256
$S_{4}^{\text{GT}}$	43.091	43.239	43.174	**42.976**	43.507
$S_{5}^{\text{GT}}$	43.401	43.010	43.497	43.773	**42.021**
MSIVA	$S_{1}^{\text{Test}}$	$S_{2}^{\text{Test}}$	$S_{3}^{\text{Test}}$	$S_{4}^{\text{Test}}$	$S_{5}^{\text{Test}}$
$S_{1}^{\text{GT}}$	**42.677**	42.865	42.751	43.038	43.111
$S_{2}^{\text{GT}}$	42.656	**42.229**	42.628	42.764	42.749
$S_{3}^{\text{GT}}$	42.695	42.862	**42.620**	43.040	43.126
$S_{4}^{\text{GT}}$	42.689	42.397	41.120	39.937	**33.609**
$S_{5}^{\text{GT}}$	43.405	42.966	43.388	43.975	**42.005**
Multimodal Baseline	$S_{1}^{\text{Test}}$	$S_{2}^{\text{Test}}$	$S_{3}^{\text{Test}}$	$S_{4}^{\text{Test}}$	$S_{5}^{\text{Test}}$
$S_{1}^{\text{GT}}$	23.824	23.947	**23.819**	24.028	24.274
$S_{2}^{\text{GT}}$	27.766	**27.442**	27.803	28.162	28.182
$S_{3}^{\text{GT}}$	23.931	24.029	**23.779**	24.036	24.229
$S_{4}^{\text{GT}}$	**17.265**	18.660	17.290	17.564	19.731
$S_{5}^{\text{GT}}$	36.764	36.359	36.758	37.265	**35.262**

**Table 2:** Neuroimaging data: Final MISA loss values (lower is better). MSIVA with the subspace structure $S_{2}$ outputs the lowest loss values in both multimodal neuroimaging datasets, thus it is considered as the optimal approach to capture the latent subspace structure in these two neuroimaging datasets. In addition, relative to the loss values in Table 1, the loss values for MSIVA are consistently lower than for the unimodal baseline, which serves as empirical evidence that MSIVA better fit these datasets.

Subspace Structure	$S_{1}$	$S_{2}$	$S_{3}$	$S_{4}$	$S_{5}$
UK Biobank Dataset					
Unimodal Baseline	47.735	47.811	47.768	47.778	47.999
MSIVA	46.794	**46.775**	46.798	46.892	46.924
Patient Dataset					
Unimodal Baseline	47.361	47.350	47.336	47.404	47.527
MSIVA	45.775	**45.674**	45.788	45.924	45.696

**Table 3:** Phenotype prediction performance using post-CCA sources from MSIVA subspace structure $S_{2}$. For the UKB dataset, sources from subspaces 5 and 4 yielded the best age regression and sex classification performance, respectively. For the patient dataset, sources from subspace 5 yielded the best age regression and diagnosis classification performance (subspace 2 performed similarly). Overall, the linked sources obtained by MSIVA $S_{2}$ show strong associations with age, sex, and SZ-related effects. Note that we estimated sources for the UKB data and the patient data independently, thus subspaces in the UKB dataset do not correspond to those in the patient dataset.

Subspace	1	2	3	4	5
UK Biobank Dataset					
Age MAE (years)	5.674	6.163	5.892	5.847	**5.378**
Sex Balanced Accuracy (%)	59.542	64.496	59.206	**79.933**	52.699
Patient Dataset					
Age MAE (years)	10.720	10.470	11.226	11.445	**10.307**
SZ-HC Diagnosis Balanced Accuracy (%)	50.565	57.624	50.000	49.691	**61.404**

**Table 4:** 54 UK Biobank phenotype variables. Variables for physical exercises in blue were reduced to 8 principal components by PCA. Variables in red were excluded in brain-age delta analysis. Variables without IDs were created by R.F.S. based on the original variables and not included in the original UK Biobank dataset.

Variable ID	Variable Name
f399 2 2	number of incorrect matches in round
f400 2 2	time to complete round
f699 2 0	length of time at current address
f864 2 0	number of daysweek walked 10 minutes
f874 2 0	duration of walks
f884 2 0	number of daysweek of moderate physical activity 10 minutes
f894 2 0	duration of moderate activity
f904 2 0	number of daysweek of vigorous physical activity 10 minutes
f914 2 0	duration of vigorous activity
f943 2 0	frequency of stair climbing in last 4 weeks
f971 2 0	frequency of walking for pleasure in last 4 weeks
f981 2 0	duration walking for pleasure
f991 2 0	frequency of strenuous sports in last 4 weeks
f1001 2 0	duration of strenuous sports
f1011 2 0	frequency of light diy in last 4 weeks
f1021 2 0	duration of light diy
f1050 2 0	time spend outdoors in summer
f1060 2 0	time spent outdoors in winter
f1070 2 0	time spent watching television tv
f1080 2 0	time spent using computer
f1160 2 0	sleep duration
f1438 2 0	bread intake
f1488 2 0	tea intake
f1498 2 0	coffee intake
f1558 2 0	alcohol intake frequency
f2139 2 0	age first had sexual intercourse
f2217 2 0	age started wearing glasses or contact lenses
f2624 2 0	frequency of heavy diy in last 4 weeks
f2634 2 0	duration of heavy diy
f3637 2 0	frequency of other exercises in last 4 weeks
f3647 2 0	duration of other exercises
f4288 2 0	time to answer
f4609 2 0	longest period of depression
f20016 2 0	fluid intelligence score
f20023 2 0	mean time to correctly identify matches
f20128 2 0	number of fluid intelligence questions attempted within time limit
f21003 2 0	age when attended assessment centre
f31 0 0	sex
	total hours walked 10 minutes
	total hours moderate physical activity 10 minutes
	total hours vigorous physical activity 10 minutes
	total hours of walking for pleasure in last 4 weeks
	total hours of strenuous sports in last 4 weeks
	total hours of other exercises in last 4 weeks
	total hours of light diy in last 4 weeks
	total hours of heavy diy in last 4 weeks
	number of physical activities wrt walking for pleasure
	years since first sexual intercourse
	years since started wearing glasses
	log time to answer
	inverse log duration screen displayed
	inverse log number of attempts
	log pm score
	fluid intelligence interaction

## Data Availability

The UK Biobank dataset can be accessed at https://www.ukbiobank.ac.uk/. The BSNIP and MPRC datasets are available through the NIMH Data Archive (NDA) https://nda.nih.gov/. The COBRE dataset is available from the Collaborative Informatics and Neuroimaging Suite (COINS) https://coins.trendscenter.org/. The FBIRN phase III dataset cannot be shared directly due to the Institutional Review Board (IRB) restrictions. Individuals interested in requesting access can contact Vince D. Calhoun, vcalhoun@gsu.edu. Analysis and visualization code for this study is publicly available at https://github.com/trendscenter/MSIVA.git. Code for brain-age delta analysis is adapted from https://www.fmrib.ox.ac.uk/datasets/BrainAgeDelta/. Code for dual-coded images is adapted from https://trendscenter.org/x/datavis/.

## Footnotes

1 We use semicolon (;) to denote that matrices are stacked vertically and comma (,) to denote that matrices are stacked horizontally.

2 The absolute values of ${\overset{ˆ}{W}}^{\left[m\right]}A^{\left[m\right]}$ entries are reported because their signs are irrelevant.

3 While a subspace is uniquely identifiable, the individual sources within each subspace are not, warranting arbitrary transformation within the subspace.

4 The modality-specific mixing matrix was estimated as the least-squares solution: ${\overset{ˆ}{A}}^{\left[m\right]}=X^{\left[m\right]}{\overset{ˆ}{S}}^{[m{]}^{⊤}}{\left({\overset{ˆ}{S}}^{\left[m\right]}{\overset{ˆ}{S}}^{[m{]}^{⊤}}\right)}^{-1}$.

5 For each voxel, we utilized singular value decomposition (SVD) of the corresponding reconstructed patterns of all modalities to capture the shared multimodal information of each cross-modal subspace.

## A Data acquisition and preprocessing

### A.1 UK Biobank dataset

#### A.1.1 Acquisition parameters

T1-weighted structural MRI (sMRI) images were acquired using a 3D MPRAGE sequence with the following parameters: repetition time (TR) = 2000ms, inversion time (TI) = 880ms, in-plane acceleration factor = 2, voxel size = 1 × 1 × 1mm^3^, acquisition matrix = 208 × 256 × 256. Resting-state functional MRI (fMRI) were acquired with the following parameters: TR = 735ms, echo time (TE) = 39ms, multiband factor = 8, in-plane acceleration factor = 1, flip angle = 52°, voxel size = 2.4 × 2.4 × 2.4mm^3^, acquisition matrix = 88 × 88 × 64.

#### A.1.2 Preprocessing steps

For sMRI preprocessing, we performed tissue segmentation and normalization to the Montreal Neurological Institute (MNI) template using the statistical parametric mapping toolbox (SPM12, http://www.fil.ion.ucl.ac.uk/spm/) (Ashburner et al., 2014), leading to gray matter (GM), white matter (WM), and cerebrospinal fluid (CSF) tissue probability maps. Next, the normalized GM tissue probability maps were spatially smoothed using a Gaussian kernel with a full width at half maximum (FWHM) = 10mm. The smoothed images were then resampled to 3 × 3 × 3mm^3^. We next defined a group mask for GM voxels. Specifically, an average GM tissue probability map from all subjects was obtained from the normalized GM tissue probability maps at 1 × 1 × 1mm^3^ resolution. This group-average GM map was binarized at a threshold of 0.2 and resampled to 3 × 3 × 3mm^3^ resolution, resulting in 44318 voxels.

For fMRI preprocessing, we utilized the distortion corrected, FIX-denoised (Griffanti et al., 2014), normalized fMRI data from the UK Biobank data resource to compute subject-specific amplitude of low frequency fluctuations (ALFF) maps, defined as the area under the low frequency band [0.01 – 0.08 Hz] power spectrum of each voxel time course in each scan. We then calculated a mean-scaled ALFF (mALFF) map for each subject, which is the subject-specific ALFF map divided by its global mean ALFF value for greater test-retest reliability (Zhao et al., 2018). The mALFF maps were smoothed using a 6mm FWHM Gaussian filter and resampled to 3 × 3 × 3mm^3^ isotropic voxels. We applied the same group-average GM mask for the mALFF maps, resulting in 44318 voxels.

### A.2 Patient datasets

#### A.2.1 Acquisition parameters

##### BSNIP.

We used the BSNIP dataset collected at two sites: 1) Baltimore with a 3-Tesla Siemens Trio Tim scanner and 2) Hartford with a 3-Tesla Siemens Allegra scanner. Isotropic T1-weighted MPRAGE scans were acquired using the following parameters: TR = 6.7ms, TE = 3.1ms, flip angle = 8°, matrix size = 256 × 240, total scan time = 10 : 52.6min, 170 sagittal slices, slice thickness = 1mm, voxel size = 1 × 1 × 1.2mm^3^ (Giakoumatos et al., 2015). Resting-state fMRI scans were obtained with the following parameters: 1) Baltimore, TR = 2210ms, TE = 30ms, flip angle = 70°, number of slices = 36, voxel size = 3.4 × 3.4 × 4mm^3^, and 140 time points; 2) Hartford, TR = 1500ms, TE = 27ms, flip angle = 70° number of slices = 29, voxel size = 3.4 × 3.4 × 5mm^3^, and 210 time points.

##### COBRE.

The COBRE dataset was collected at a single site using a 3-Tesla Siemens Tim Trio scanner. A high-resolution T1-weighted multi-echo MPRAGE sequence was used with the following parameters: TR = 2530ms, TE = [1.64, 3.5, 5.36, 7.22, 9.08]ms, TI = 900ms, flip angle = 7°, acquisition matrix = 256 × 256 × 176, voxel size = 1 × 1 × 1mm^3^, number of echos = 5, pixel bandwidth = 650Hz, total scan time = 6min. Resting-state fMRI scans were collected with a standard single-shot full k-space echo-planar imaging (EPI) sequence: TR = 2000ms, TE = 29ms, voxel size = 3.75 × 3.75 × 4.55mm^3^, slice gap = 1.05mm, flip angle = 75°, number of slices = 32, field of view (FOV) = 240 × 240mm^2^, matrix size = 64 × 64, and 149 volumes. See https://fcon_1000.projects.nitrc.org/indi/retro/cobre.html for more details.

##### FBIRN.

The FBIRN phase III dataset was collected from seven sites. Out of seven sites, six sites used 3-Tesla Siemens Tim Trio scanners and one site used a 3-Tesla General Electric (GE) Discovery MR750 scanner. A high-resolution Siemens MPRAGE sequence was acquired with the following parameters: TR/TE/TI = 2300/2.94/1100ms, flip angle = 9°, acquisition matrix = 256 × 256 × 160. Likewise, a GE IR-SPGR sequence was acquired with the following parameters: TR/TE/TI = 5.95/1.99/45ms, flip angle = 12°, acquisition matrix = 256 × 256 × 166, FOV = 220 × 220mm^2^, voxel size = 0.86 × 0.86 × 1.2mm^3^, collected in the sagittal plane with GRAPPA/ASSET acceleration factor = 2, and NEX = 1 (Qi et al., 2022). The same resting-state fMRI parameters were used across all seven sites: a standard gradient EPI sequence, TR/TE = 2000/30ms, voxel size = 3.4375 × 3.4375 × 4mm^3^, slice gap = 1mm, flip angle = 77°, FOV = 220 × 220mm^2^, and 162 volumes (Qi et al., 2022).

##### MPRC.

The MPRC dataset was collected at three sites, each using a different 3-Tesla Siemens scanner, with a standard EPI sequence. T1-weighted 3D MPRAGE sequence was collected in the saggital plane with voxel size = 1 × 1 × 1mm^3^ using a Siemens Allegra scanner (TE/TR/TI = 4.3/2500/1000ms, flip angle = 8°) or a Siemens Trio scanner (TE/TR/TI = 2.9/2300/900ms, flip angle = 9°) (Schijven et al., 2023). Resting-state fMRI scans were collected using the following scanners and parameters: 3-Tesla Siemens Allegra scanner (TR/TE = 2000/27ms, voxel size = 3.44 × 3.44 × 4mm^3^, FOV = 220 × 220mm^2^, and 150 volumes); 3-Tesla Siemens Trio scanner (TR/TE = 2210/30ms, voxel size = 3.44 × 3.44 × 4mm^3^, FOV = 220 × 220mm^2^, and 140 volumes); and 3-Tesla Siemens Tim Trio scanner (TR/TE = 2000/30ms, voxel size = 1.72 × 1.72 × 4mm^3^, FOV = 220 × 220mm^2^, and 444 volumes) (Qi et al., 2022).

#### A.2.2 Preprocessing steps

All sMRI datasets were preprocessed using SPM12, following the steps described in Qi et al., 2022. Specifically, the data were normalized to the MNI template using unified segmentation, resampled to 3 × 3 × 3mm^3^, and segmented into GM, WM, and CSF using modulated normalization, leading to GM volume maps. These GM volume maps were then smoothed using a 6mm FWHM Gaussian kernel. To ensure proper segmentation for all subjects, outlier detection was performed using spatial Pearson correlation with the template image.

All fMRI datasets underwent the preprocessing steps as outlined in Qi et al., 2022: removal of the initial five scans to eliminate T1 equilibration effects, slice timing correction, realignment, normalization to the EPI template with 3 × 3 × 3mm^3^ resolution, spatial smoothing using a 6mm FWHM Gaussian kernel, regression of nuisance covariates (including six head motion parameters, CSF, WM) and global signal from the voxelwise time course using a general linear model, and computation of the mALFF maps.

## B Voxelwise brain-age delta analysis on UK Biobank data

We performed a voxelwise brain-age delta analysis using the estimated sources $\overset{ˆ}{S}$ from MSIVA subspace structure $S_{2}$ in the UK Biobank dataset. We describe the steps to construct imaging-derived predictors as follows.

1. Reconstruction. We first reconstructed modality- and subspace-specific imaging feature ${\overset{ˆ}{X}}_{k}^{\left[m\right]}={\overset{ˆ}{A}}_{k}^{\left[m\right]}{\overset{ˆ}{S}}_{k}^{\left[m\right]}$ for each of five multimodal subspaces $\left({\overset{ˆ}{A}}_{k}^{\left[m\right]}\in R^{V\times 2},{\overset{ˆ}{S}}_{k}^{\left[m\right]}\in R^{2\times N},k\in \{1,\ldots ,5\}\right)$ and each of four unimodal subspaces $\left({\hat{A}}_{k}^{\left[m\right]}\in {\mathbb{R}}^{V\times 1},{\hat{S}}_{k}^{\left[m\right]}\in {\mathbb{R}}^{1\times N},k\in \left\{6,\ldots ,9\right\}\right)$, where k is the subspace index. Here, subspaces 6 and 7 are used exclusively for sMRI and subspaces 8 and 9 are used exclusively for fMRI.
2. Singular value decomposition. For each voxel i in the reconstructed imaging data ${\overset{ˆ}{X}}_{k[i,:]}^{\left[m\right]}\in R^{N}$ from each of five cross-modal subspaces, we concatenated the two modalities ${\overset{ˆ}{X}}_{k[i,:]}=\left[{\overset{ˆ}{X}}_{k[i,:]}^{\left[1\right]},{\overset{ˆ}{X}}_{k[i,:]}^{\left[2\right]}\right]{,\overset{ˆ}{X}}_{k[i,:]}\in R^{N\times 2}$, normalized ${\overset{ˆ}{X}}_{k[i,:]}$ along the rows^6^, and then performed singular value decomposition (SVD) on ${\overset{ˆ}{X}}_{k[i,:]}$, i.e. ${\overset{ˆ}{X}}_{k[i,:]}=U\Sigma V^{⊤}$. Next, we multiplied ${\overset{ˆ}{X}}_{k[i,:]}$ by the first left singular vector $V_{\left[:,1\right]}\in R^{2\times 1}$ corresponding to the largest singular value $\lambda_{\text{max}}$, leading to ${\overset{ˆ}{X}}_{k[i,:]}^{\text{SVD}}={\overset{ˆ}{X}}_{k[i,:]}V_{\left[:,1\right]},\;{\overset{ˆ}{X}}_{k[i,:]}^{\text{SVD}}\in R^{N\times 1}$. We then normalized ${\overset{ˆ}{X}}_{k[i,:]}^{\text{SVD}}$ to obtain the normalized ${\hat{X}}_{k\left[i,:\right]}^{\text{SV}{\text{D}}^{\prime }}$.
3. Partialization and normalization. We next partialized and normalized ${\hat{X}}_{k\left[i,:\right]}^{\text{SV}{\text{D}}^{\prime }}$ and ${\overset{ˆ}{X}}_{k[i,:]}^{\left[m\right]}$ (all five cross-modal subspaces in sMRI and the four unimodal subspaces in both modalities) to remove SVD-related confounds from ${\overset{ˆ}{X}}_{k[i,:]}^{\left[m\right]}$, leading to ${\overset{ˆ}{X}}_{k[i,:]}^{[m{]}^{\prime }}$.
4. Concatenation. We concatenated the SVD results from Step 2 (without partialization or extra normalization) ${\overset{ˆ}{X}}_{k[i,:]}^{\text{SV}{\text{D}}^{\prime }}$ from five cross-modal subspaces, and modality-specific partialized and normalized ${\overset{ˆ}{X}}_{k[i,:]}^{[m{]}^{\prime }}$ from five cross-modal subspaces and four unimodal subspaces, resulting in 14 predictors in total, ${\overset{ˆ}{X}}_{i}=\left[{\overset{ˆ}{X}}_{1[i,:]}^{\text{SV}{\text{D}}^{\prime }},\ldots ,{\overset{ˆ}{X}}_{5[i,:]}^{\text{SV}{\text{D}}^{\prime }},{\overset{ˆ}{X}}_{1[i,:]}^{[1{]}^{\prime }},\ldots ,{\overset{ˆ}{X}}_{7[i,:]}^{[1{]}^{\prime }},{\overset{ˆ}{X}}_{8[i,:]}^{[2{]}^{\prime }},{\overset{ˆ}{X}}_{9[i,:]}^{[2{]}^{\prime }}\right],{\overset{ˆ}{X}}_{i}\in R^{N\times 14}$.

For each voxel i, we performed a two-stage brain age prediction where the first stage estimates the initial delta and the second stage further removes age dependence and other confound factors from the delta (Smith et al., 2019, 2020):

(12)
$$\delta_{1}={\hat{X}}_{i}\beta_{1}-y,$$

(13)
$$\delta_{2}=\delta_{1}-Y\beta_{2},$$

where $y\in R^{N}$ is the chronological age after removing the mean age across subjects. $Y\in R^{N\times 10}$ includes the confound variables:

1. the demeaned linear age term,
2. the demeaned quadratic age term after regressing out the linear age effects and normalizing to have the same standard deviation as the linear age term,
3. the demeaned cubic age term after regressing out the linear and quadratic age effects and normalizing to have the same standard deviation as the linear age term,
4. sex,
5. the interaction between sex and each of the three age terms,
6. the framewise displacement variable, and
7. the spatial normalization variables from sMRI and FMRI.

Finally, we partialized $\delta_{2}$ to remove residual associations, obtaining the partialized brain-age delta, $\delta_{2p}$.

## C UK Biobank phenotype variables

We used 25 phenotype variables, including lifestyle measures and cognitive test scores, to investigate their associations with brain-age delta. We describe the process of selecting the phenotype variables as follows.

We first excluded variables with extreme values from original 64 non-imaging variables using a two-step approach:

1. We calculated the sum of squared absolute median deviations (d) for each variable.
2. We excluded any variable where max(d)>100×mean(d), as these extreme outliers could skew statistical analysis.

This initial screening resulted in 54 variables, including age, sex, fluid intelligence, physical activity measures, alcohol intake frequency, cognitive test scores, time spent watching TV, and sleep duration. We further reduced or excluded phenotype variables from these 54 variables as described below:

1. We applied PCA to decompose 28 physical exercise variables into 8 principal components.
2. We removed five age-related variables due to high correlation with other age variables. These variables were “age when attended assessment center”, “age when first sexual intercourse”, “age started wearing glasses”, “years since first sexual intercourse”, and “years since started wearing glasses”.
3. We excluded two variables related to a cognitive test (“time to answer” and “log time to answer”) due to distinct population distributions resulting from two different cognitive tests used during data collection.
4. Finally, we removed the sex variable and another log variable (“log pm score”).

This selection process ultimately yielded 25 variables in total for our analysis, as listed in Table 4.

## D Nonlinear source dependence in neuroimaging data

Apart from Pearson correlation, we calculated the randomized correlation coefficents (RDCs) (Lopez-Paz et al., 2013) to measure nonlinear source dependence in neuroimaging data. The RDC results (Figures 14,15) are largely consistent with those measured by Pearson correlation (Figures 5, 6). The low RDC values outside the block subspace structure indicate very weak residual dependence between subspaces, suggesting that different subspaces are nearly independent.

## E MSIVA $S_{2}$ reconstructed neuroimaging data

Figure 16 shows spatial maps of group-specific reconstructed data from each of five MSIVA $S_{2}$ linked subspaces in the UKB dataset. Similarly, Figure 17 shows results related to the age and SZ interaction effects in the patient dataset. In each panel, axial slices show the geometric median of the reconstructed subspace data $\left({\overset{ˆ}{X}}_{k}^{\left[m\right]}\right)$ for each modality and each group. Voxel intensity is mapped to both color hue and opacity. The contours highlight the brain areas where voxelwise cross-modal correlations are significant for each group (P<0.01, Bonferroni correction for 44318 voxels). Scatter plots show post-CCA sources color-coded by age, sex, or diagnosis label. The reported $R^{2}$ indicates the proportion of variance captured by the subspace in each modality.

Figure 18 illustrates the number of voxels that show significant cross-modal correlations for age and sex groups in the UKB dataset (rows I and II), and for age and diagnosis groups in the patient dataset (rows III and IV). We find that the number of voxels for older patients diagnosed with SZ is consistently less than that for their age-matched control subjects in four of five subspaces, implying reduced brain structure-function coupling in the older patient group.

## F Comparison between MSIVA $S_{2}$ and MMIVA sources

MSIVA can be viewed as an extension of MMIVA with two main differences. First, MSIVA uses a flexible *block* diagonal subspace structure while MMIVA uses a rigid identity matrix as the subspace structure. Second, MSIVA uses MGPCA and separate ICAs initialization while MMIVA uses MGPCA and group ICA initialization. To further investigate similarities and differences of the recovered sources from MSIVA and MMIVA, we compared MSIVA with the subspace structure $S_{2}$ and MMIVA with the subspace structure $S_{5}$ through the following experiments:

1. We performed multiple linear regression (MLR) for each modality using MSIVA $S_{2}$ post-CCA sources from each cross-modal subspace $X_{i}^{\left[m\right]}$ to predict each MMIVA source $y_{j}^{\left[m\right]}$:
  (14)
  $$y_{j}^{\left[m\right]}=X_{i}^{\left[m\right]}\beta ,$$
  where i∈{1,…,5} is the cross-modal subspace index in MSIVA $S_{2}$, and j∈{1,…,12} is the subspace index in MMIVA.
2. We performed multivariate analysis of variance (MANOVA) for each modality using a pair of matched MMIVA sources $\left[y_{j}^{\left[m\right]},y_{k}^{\left[m\right]}\right]$ from Step 1 to predict MSIVA $S_{2}$ post-CCA sources from each cross-modal subspace $X_{i}^{\left[m\right]}$:
  (15)
  $$X_{i}^{\left[m\right]}=\left[y_{j}^{\left[m\right]},y_{k}^{\left[m\right]}\right]\beta .$$
  Here, (j,k) are a pair of matched subspace indices in MMIVA, and i is the cross-modal subspace index in MSIVA $S_{2}$.

We measured the adjusted $R^{2}\left(R_{adj}^{2}\right)$ from MLR as shown below:

(16)
$$R^{2}=1-\frac{\sum_{i=1}^{N}{\left(y_{i}-{\hat{y}}_{i}\right)}^{2}}{\sum_{i=1}^{N}{\left(y_{i}-\bar{y}\right)}^{2}},\;\bar{y}=\frac{1}{N}\overset{N}{\underset{i=1}{\sum }}y_{i},$$

(17)
$$R_{adj}^{2}=1-\left(1-R^{2}\right)\frac{N-1}{N-N_{P}-1},$$

where N is the number of samples (here subjects) and $N_{P}$ is the number of predictors.

Figures 19 and 21 show the adjusted $R^{2}$ when using pairs of MSIVA $S_{2}$ sources from each cross-modal subspace to predict each of the 12 MMIVA sources for the UKB dataset and the patient dataset, respectively. We reordered MMIVA sources to identify the most likely correspondence between MSIVA $S_{2}$ sources and MMIVA sources. We notice that there exists some correspondence between MSIVA $S_{2}$ sources and MMIVA sources. For example, for UKB sMRI data, MSIVA $S_{2}$ subspace 3 sources match MMIVA source $3\left(R_{adj}^{2}=0.96\right)$, MSIVA $S_{2}$ subspace 5 sources match MMIVA source $5\left(R_{adj}^{2}=0.87\right)$, and MSIVA $S_{2}$ subspace 4 sources match MMIVA source $2\left(R_{adj}^{2}=0.73\right)$. We also observe that there are more than two columns showing high $R_{adj}^{2}(>0.2)$ for each row, indicating that every two MSIVA $S_{2}$ sources from each subspace can predict variability for more than two MMIVA sources. Note that the prediction results for fMRI are very consistent with those for sMRI.

We then performed MANOVA using every two matched MMIVA sources to predict the pair of MSIVA $S_{2}$ sources from each cross-modal subspace. We show the Pillai’s trace divided by the number of modalities (M=2) in Figures 20 and 22. Note that the maximum possible diagonal Pillai’s trace value is 2 for two modalities, and dividing the Pillai’s trace by 2 shows the variance explained for each modality. We observe large off-diagonal values per column, indicating that every pair of matched MMIVA sources predicts variability of more than two MSIVA $S_{2}$ sources. Note that the prediction results for fMRI are very consistent with those for sMRI.

Therefore, we conclude that MSIVA and MMIVA apportion variability to their sources in different ways. There is no perfect one-to-one mapping between MSIVA $S_{2}$ sources and MMIVA sources. We also note that the mismatch appears to be more pronounced in the patient dataset than in the UKB dataset, which may be related to inherent characteristics of the patient data, such as higher population heterogeneity and smaller sample size.

## References

[R1] Adali T., Anderson M., & Fu G.-S. (2014). Diversity in independent component and vector analyses: Identifiability, algorithms, and applications in medical imaging. IEEE Signal Processing Magazine, 31(3), 18–33.

[R2] Adali T., Levin-Schwartz Y., & Calhoun V. D. (2015a). Multimodal data fusion using source separation: Application to medical imaging. Proceedings of the IEEE, 103(9), 1494–1506. 10.1109/JPROC.2015.2461624 PMC4624202 26525830

[R3] Adali T., Levin-Schwartz Y., & Calhoun V. D. (2015b). Multimodal data fusion using source separation: Two effective models based on ica and iva and their properties. Proceedings of the IEEE, 103(9), 1478–1493. 26525830 10.1109/JPROC.2015.2461624 PMC4624202

[R4] Aine C., Bockholt H. J., Bustillo J. R., Cañive J. M., Caprihan A., Gasparovic C., Hanlon F. M., Houck J. M., Jung R. E., Lauriello J., (2017). Multimodal neuroimaging in schizophrenia: Description and dissemination. Neuroinformatics, 15(4), 343–364. 28812221 10.1007/s12021-017-9338-9 PMC5671541

[R5] Allen E. A., Erhardt E. B., & Calhoun V. D. (2012). Data visualization in the neurosciences: Overcoming the curse of dimensionality. Neuron, 74(4), 603–608. 22632718 10.1016/j.neuron.2012.05.001 PMC4427844

[R6] Amari S.-I., Cichocki A., & Yang H. H. (1996). A New Learning Algorithm for Blind Signal Separation. Proc NIPS 1996, 8, 757–763.

[R7] Ashburner J., Barnes G., Chen C.-C., Daunizeau J., Flandin G., Friston K., Kiebel S., Kilner J., Litvak V., Moran R., (2014). Spm12 manual. Wellcome Trust Centre for Neuroimaging, London, UK, 2464(4).

[R8] Bao P., She L., McGill M., & Tsao D. Y. (2020). A map of object space in primate inferotemporal cortex. Nature, 583(7814), 103–108. 32494012 10.1038/s41586-020-2350-5 PMC8088388

[R9] Bell A. J., & Sejnowski T. J. (1995). An information-maximization approach to blind separation and blind deconvolution. Neural computation, 7(6), 1129–1159. 7584893 10.1162/neco.1995.7.6.1129

[R10] Bernardi S., Benna M. K., Rigotti M., Munuera J., Fusi S., & Salzman C. D. (2020). The geometry of abstraction in the hippocampus and prefrontal cortex. Cell, 183(4), 954–967. 33058757 10.1016/j.cell.2020.09.031 PMC8451959

[R11] Boyle L. M., Posani L., Irfan S., Siegelbaum S. A., & Fusi S. (2024). Tuned geometries of hippocampal representations meet the computational demands of social memory. Neuron, 112(8), 1358–1371. 38382521 10.1016/j.neuron.2024.01.021 PMC11186585

[R12] Calhoun V. D., & Adali T. (2008). Feature-based fusion of medical imaging data. IEEE Transactions on Information Technology in Biomedicine, 13(5), 711–720. 19273016 10.1109/TITB.2008.923773 PMC2737598

[R13] Calhoun V. D., Adali T., Giuliani N., Pekar J., Kiehl K., & Pearlson G. (2006). Method for multimodal analysis of independent source differences in schizophrenia: Combining gray matter structural and auditory oddball functional data. Human brain mapping, 27(1), 47–62. 16108017 10.1002/hbm.20166 PMC6871470

[R14] Calhoun V. D., Adali T., Pearlson G. D., & Kiehl K. A. (2006). Neuronal chronometry of target detection: Fusion of hemodynamic and event-related potential data. Neuroimage, 30(2), 544–553. 16246587 10.1016/j.neuroimage.2005.08.060

[R15] Calhoun V. D., & Sui J. (2016). Multimodal fusion of brain imaging data: A key to finding the missing link (s) in complex mental illness. Biological psychiatry: cognitive neuroscience and neuroimaging, 1(3), 230–244. 27347565 10.1016/j.bpsc.2015.12.005 PMC4917230

[R16] Cardoso J.-F. (1998). Multidimensional independent component analysis. Proceedings of the 1998 IEEE International Conference on Acoustics, Speech and Signal Processing, ICASSP’98 (Cat. No. 98CH36181), 4, 1941–1944.

[R17] Chang L., & Tsao D. Y. (2017). The code for facial identity in the primate brain. Cell, 169(6), 1013–1028. 28575666 10.1016/j.cell.2017.05.011 PMC8088389

[R18] Churchland M. M., Cunningham J. P., Kaufman M. T., Foster J. D., Nuyujukian P., Ryu S. I., & Shenoy K. V. (2012). Neural population dynamics during reaching. Nature, 487(7405), 51–56. 22722855 10.1038/nature11129 PMC3393826

[R19] Comon P. (1994). Independent component analysis, a new concept? Signal processing, 36(3), 287–314.

[R20] Correa N. M., Adali T., Li Y.-O., & Calhoun V. D. (2010). Canonical correlation analysis for data fusion and group inferences. IEEE signal processing magazine, 27(4), 39–50. 20706554 10.1109/MSP.2010.936725 PMC2919827

[R21] Correa N. M., Li Y.-O., Adali T., & Calhoun V. D. (2008). Canonical correlation analysis for feature-based fusion of biomedical imaging modalities and its application to detection of associative networks in schizophrenia. IEEE journal of selected topics in signal processing, 2(6), 998–1007. 19834573 10.1109/JSTSP.2008.2008265 PMC2761661

[R22] Courellis H. S., Minxha J., Cardenas A. R., Kimmel D. L., Reed C. M., Valiante T. A., Salzman C. D., Mamelak A. N., Fusi S., & Rutishauser U. (2024). Abstract representations emerge in human hippocampal neurons during inference. Nature, 1–9. 10.1038/s41586-024-07799-x PMC11338822 39143207

[R23] Du Y., Fu Z., Sui J., Gao S., Xing Y., Lin D., Salman M., Abrol A., Rahaman M. A., Chen J., (2020). Neuromark: An automated and adaptive ica based pipeline to identify reproducible fmri markers of brain disorders. Neurolmage: Clinical, 28, 102375. 10.1016/j.nicl.2020.102375 PMC7509081 32961402

[R24] Fan L., Tang Y., Sun B., Gong G., Chen Z. J., Lin X., Yu T., Li Z., Evans A. C., & Liu S. (2010). Sexual dimorphism and asymmetry in human cerebellum: An mri-based morphometric study. Brain research, 1353, 60–73. 20647004 10.1016/j.brainres.2010.07.031

[R25] Franco A. R., Ling J., Caprihan A., Calhoun V. D., Jung R. E., Heileman G. L., & Mayer A. R. (2008). Multimodal and multi-tissue measures of connectivity revealed by joint independent component analysis. IEEE journal of selected topics in signal processing, 2(6), 986–997. 19777078 10.1109/JSTSP.2008.2006718 PMC2748354

[R26] Fu Z., Batta I., Wu L., Abrol A., Agcaoglu O., Salman M. S., Du Y., Iraji A., Shultz S., Sui J., (2024). Searching reproducible brain features using neuromark: Templates for different age populations and imaging modalities. Neurolmage, 292, 120617. 10.1016/j.neuroimage.2024.120617 PMC11416721 38636639

[R27] Giakoumatos C., Nanda P., Mathew I., Tandon N., Shah J., Bishop J., Clementz B., Pearlson G., Sweeney J., Tamminga C., (2015). Effects of lithium on cortical thickness and hippocampal subfield volumes in psychotic bipolar disorder. Journal of psychiatric research, 61, 180–187. 25563516 10.1016/j.jpsychires.2014.12.008 PMC4859940

[R28] Griffanti L., Salimi-Khorshidi G., Beckmann C. F., Auerbach E. J., Douaud G., Sexton C. E., Zsoldos E., Ebmeier K. P., Filippini N., Mackay C. E., (2014). Ica-based artefact removal and accelerated fmri acquisition for improved resting state network imaging. Neuroimage, 95, 232–247. 24657355 10.1016/j.neuroimage.2014.03.034 PMC4154346

[R29] Groves A. R., Beckmann C. F., Smith S. M., & Woolrich M. W. (2011). Linked independent component analysis for multimodal data fusion. Neuroimage, 54(3), 2198–2217. 20932919 10.1016/j.neuroimage.2010.09.073

[R30] Hajnal M. A., Tran D., Szabó Z., Albert A., Safaryan K., Einstein M., Vallejo Martelo M., Polack P.-O., Golshani P., & Orbán G. (2024). Shifts in attention drive context-dependent subspace encoding in anterior cingulate cortex in mice during decision making. Nature communications, 15(1), 5559. 10.1038/s41467-024-49845-2 PMC11220070 38956080

[R31] Hogstrom L. J., Westlye L. T., Walhovd K. B., & Fjell A. M. (2013). The structure of the cerebral cortex across adult life: Age-related patterns of surface area, thickness, and gyrification. Cerebral cortex, 23(11), 2521–2530. 22892423 10.1093/cercor/bhs231

[R32] Hotelling H. (1992). Relations between two sets of variates. In Breakthroughs in statistics (pp. 162–190). Springer.

[R33] Jernigan T. L., Archibald S. L., Fennema-Notestine C., Gamst A. C., Stout J. C., Bonner J., & Hesselink J. R. (2001). Effects of age on tissues and regions of the cerebrum and cerebellum. Neurobiology of aging, 22(4), 581–594. 11445259 10.1016/s0197-4580(01)00217-2

[R34] Johnston W. J., Fine J. M., Yoo S. B. M., Ebitz R. B., & Hayden B. Y. (2024). Semi-orthogonal subspaces for value mediate a binding and generalization trade-off. Nature Neuroscience, 1–13. 10.1038/s41593-024-01758-5 PMC12063212 39289564

[R35] Keator D. B., van Erp T. G., Turner J. A., Glover G. H., Mueller B. A., Liu T. T., Voyvodic J. T., Rasmussen J., Calhoun V. D., Lee H. J., (2016). The function biomedical informatics research network data repository. Neuroimage, 124, 1074–1079. 26364863 10.1016/j.neuroimage.2015.09.003 PMC4651841

[R36] Kim T., Eltoft T., & Lee T.-W. (2006). Independent vector analysis: An extension of ica to multivariate components. International conference on independent component analysis and signal separation, 165–172.

[R37] Kotz S. (1975). Multivariate distributions at a cross road. In A modern course on statistical distributions in scientific work (pp. 247–270). Springer.

[R38] Lahat D., Adali T., & Jutten C. (2015). Multimodal data fusion: An overview of methods, challenges, and prospects. Proceedings of the IEEE, 103(9), 1449–1477.

[R39] Li X., Adali T., Silva R. F., & Calhoun V. D. (2023). Multimodal subspace independent vector analysis better captures hidden relationships in multimodal neuroimaging data. 2023 IEEE 20th International Symposium on Biomedical Imaging (ISBI), 1–5.

[R40] Liu D. C., & Nocedal J. (1989). On the limited memory bfgs method for large scale optimization. Mathematical programming, 45(1–3), 503–528.

[R41] Lopez-Paz D., Hennig P., & Schölkopf B. (2013). The randomized dependence coefficient. Advances in neural information processing systems, 26.

[R42] Luft A. R., Skalej M., Schulz J. B., Welte D., Kolb R., Bürk K., Klockgether T., & Voigt K. (1999). Patterns of age-related shrinkage in cerebellum and brainstem observed in vivo using three-dimensional mri volumetry. Cerebral Cortex, 9(7), 712–721. 10554994 10.1093/cercor/9.7.712

[R43] Ma S., Correa N. M., Li X.-L., Eichele T., Calhoun V. D., & Adali T. (2011). Automatic identification of functional clusters in fmri data using spatial dependence. IEEE Transactions on Biomedical Engineering, 58(12), 3406–3417. 21900068 10.1109/TBME.2011.2167149 PMC3222740

[R44] Ma S., Li X.-L., Correa N. M., Adali T., & Calhoun V. D. (2010). Independent subspace analysis with prior information for fmri data. 2010 IEEE International Conference on Acoustics, Speech and Signal Processing, 1922–1925.

[R45] Macchi O., & Moreau E. (1995). Self-adaptive source separation by direct or recursive networks. Proc ICDSP 1995, 122–129.

[R46] Martínez A., Hillyard S. A., Dias E. C., Hagler D. J., Butler P. D., Guilfoyle D. N., Jalbrzikowski M., Silipo G., & Javitt D. C. (2008). Magnocellular pathway impairment in schizophrenia: Evidence from functional magnetic resonance imaging. Journal of Neuroscience, 28(30), 7492–7500. 18650327 10.1523/JNEUROSCI.1852-08.2008 PMC6670855

[R47] Miller K. L., Alfaro-Almagro F., Bangerter N. K., Thomas D. L., Yacoub E., Xu J., Bartsch A. J., Jbabdi S., Sotiropoulos S. N., Andersson J. L., (2016). Multimodal population brain imaging in the uk biobank prospective epidemiological study. Nature neuroscience, 19(11), 1523–1536. 27643430 10.1038/nn.4393 PMC5086094

[R48] Moberget T., Doan N., Alnæs D., Kaufmann T., Córdova-Palomera A., Lagerberg T., Diedrichsen J., Schwarz E., Zink M., Eisenacher S., (2018). Cerebellar volume and cerebellocerebral structural covariance in schizophrenia: A multisite mega-analysis of 983 patients and 1349 healthy controls. Molecular psychiatry, 23(6), 1512–1520. 28507318 10.1038/mp.2017.106

[R49] Mohammadi-Nejad A.-R., Hossein-Zadeh G.-A., & Soltanian-Zadeh H. (2017). Structured and sparse canonical correlation analysis as a brain-wide multi-modal data fusion approach. IEEE transactions on medical imaging, 36(7), 1438–1448. 28320654 10.1109/TMI.2017.2681966

[R50] Pandarinath C., O’Shea D. J., Collins J., Jozefowicz R., Stavisky S. D., Kao J. C., Trautmann E. M., Kaufman M. T., Ryu S. I., Hochberg L. R., (2018). Inferring single-trial neural population dynamics using sequential auto-encoders. Nature methods, 15(10), 805–815. 30224673 10.1038/s41592-018-0109-9 PMC6380887

[R51] Picard H., Amado I., Mouchet-Mages S., Olié J.-P., & Krebs M.-O. (2008). The role of the cerebellum in schizophrenia: An update of clinical, cognitive, and functional evidences. Schizophrenia bulletin, 34(1), 155–172. 17562694 10.1093/schbul/sbm049 PMC2632376

[R52] Qi S., Sui J., Pearlson G., Bustillo J., Perrone-Bizzozero N. I., Kochunov P., Turner J. A., Fu Z., Shao W., Jiang R., Yang X., Liu J., Du Y., Chen J., Zhang D., & Calhoun V. D. (2022). Derivation and utility of schizophrenia polygenic risk associated multimodal MRI frontotemporal network. Nat Commun, 13(1), 4929. 10.1038/s41467-022-32513-8 35995794 PMC9395379

[R53] Remington E. D., Narain D., Hosseini E. A., & Jazayeri M. (2018). Flexible sensorimotor computations through rapid reconfiguration of cortical dynamics. Neuron, 98(5), 1005–1019. 29879384 10.1016/j.neuron.2018.05.020 PMC6009852

[R54] Romero J. E., Coupe P., Lanuza E., Catheline G., Manjón J. V., & Initiative A. D. N. (2021). Toward a unified analysis of cerebellum maturation and aging across the entire lifespan: A mri analysis. Human Brain Mapping, 42(5), 1287–1303. 33385303 10.1002/hbm.25293 PMC7927303

[R55] Ruigrok A. N., Salimi-Khorshidi G., Lai M.-C., Baron-Cohen S., Lombardo M. V., Tait R. J., & Suckling J. (2014). A meta-analysis of sex differences in human brain structure. Neuroscience & Biobehavioral Reviews, 39, 34–50. 10.1016/j.neubiorev.2013.12.004 PMC3969295 24374381

[R56] Scheinost D., Finn E. S., Tokoglu F., Shen X., Papademetris X., Hampson M., & Constable R. T. (2015). Sex differences in normal age trajectories of functional brain networks. Human brain mapping, 36(4), 1524–1535. 25523617 10.1002/hbm.22720 PMC5522589

[R57] Schijven D., Postema M. C., Fukunaga M., Matsumoto J., Miura K., de Zwarte S. M., Van Haren N. E., Cahn W., Hulshoff Pol H. E., Kahn R. S., (2023). Large-scale analysis of structural brain asymmetries in schizophrenia via the enigma consortium. Proceedings of the National Academy of Sciences, 120(14), e2213880120. 10.1073/pnas.2213880120 PMC10083554 36976765

[R58] Semedo J. D., Zandvakili A., Machens C. K., Byron M. Y., & Kohn A. (2019). Cortical areas interact through a communication subspace. Neuron, 102(1), 249–259. 30770252 10.1016/j.neuron.2019.01.026 PMC6449210

[R59] She L., Benna M. K., Shi Y., Fusi S., & Tsao D. Y. (2024). Temporal multiplexing of perception and memory codes in it cortex. Nature, 1–8. 10.1038/s41586-024-07349-5 PMC11111405 38750353

[R60] SilvaR. F., DamarajuE., LiX., KochunovP., BelgerA., FordJ. M., MathalonD. H., MuellerB. A., PotkinS. G., PredaA., (2021). Direct linkage detection with multimodal iva fusion reveals markers of age, sex, cognition, and schizophrenia in large neuroimaging studies. bioRxiv, 2021–12.10.1002/hbm.7003739560198

[R61] Silva R. F., Plis S. M., Adalı T., Pattichis M. S., & Calhoun V. D. (2020). Multidataset independent subspace analysis with application to multimodal fusion. IEEE Transactions on Image Processing, 30, 588–602. 33031036 10.1109/TIP.2020.3028452 PMC7877797

[R62] Smith S. M., Elliott L. T., Alfaro-Almagro F., McCarthy P., Nichols T. E., Douaud G., & Miller K. L. (2020). Brain aging comprises many modes of structural and functional change with distinct genetic and biophysical associations. elife, 9, e52677. 32134384 10.7554/eLife.52677 PMC7162660

[R63] Smith S. M., Nichols T. E., Vidaurre D., Winkler A. M., Behrens T. E., Glasser M. F., Ugurbil K., Barch D. M., Van Essen D. C., & Miller K. L. (2015). A positive-negative mode of population covariation links brain connectivity, demographics and behavior. Nature neuroscience, 18(11), 1565–1567. 26414616 10.1038/nn.4125 PMC4625579

[R64] Smith S. M., Vidaurre D., Alfaro-Almagro F., Nichols T. E., & Miller K. L. (2019). Estimation of brain age delta from brain imaging. Neuroimage, 200, 528–539. 31201988 10.1016/j.neuroimage.2019.06.017 PMC6711452

[R65] Song Y., Schreier P. J., Ramírez D., & Hasija T. (2016). Canonical correlation analysis of high-dimensional data with very small sample support. Signal Processing, 128, 449–458.

[R66] Sui J., Adali T., Yu Q., Chen J., & Calhoun V. D. (2012). A review of multivariate methods for multimodal fusion of brain imaging data. Journal of neuroscience methods, 204(1), 68–81. 22108139 10.1016/j.jneumeth.2011.10.031 PMC3690333

[R67] Sui J., He H., Pearlson G. D., Adali T., Kiehl K. A., Yu Q., Clark V. P., Castro E., White T., Mueller B. A., (2013). Three-way (n-way) fusion of brain imaging data based on mcca+jica and its application to discriminating schizophrenia. Neurolmage, 66, 119–132. 10.1016/j.neuroimage.2012.10.051 PMC3897558 23108278

[R68] Sui J., Pearlson G., Caprihan A., Adali T., Kiehl K. A., Liu J., Yamamoto J., & Calhoun V. D. (2011). Discriminating schizophrenia and bipolar disorder by fusing fmri and dti in a multimodal cca+ joint ica model. Neuroimage, 57(3), 839–855. 21640835 10.1016/j.neuroimage.2011.05.055 PMC3129373

[R69] Szabó Z., Póczos B., & L \Horincz A. (2012). Separation theorem for independent subspace analysis and its consequences. Pattern Recognit, 45(4), 1782–1791. 10.1016/j.patcog.2011.09.007

[R70] Tamminga C. A., Pearlson G., Keshavan M., Sweeney J., Clementz B., & Thaker G. (2014). Bipolar and schizophrenia network for intermediate phenotypes: Outcomes across the psychosis continuum. Schizophrenia bulletin, 40(Suppl_2), S131–S137. 24562492 10.1093/schbul/sbt179 PMC3934403

[R71] Tian L., Wang J., Yan C., & He Y. (2011). Hemisphere-and gender-related differences in small-world brain networks: A resting-state functional mri study. Neuroimage, 54(1), 191–202. 20688177 10.1016/j.neuroimage.2010.07.066

[R72] Wang J., Narain D., Hosseini E. A., & Jazayeri M. (2018). Flexible timing by temporal scaling of cortical responses. Nature neuroscience, 21(1), 102–110. 29203897 10.1038/s41593-017-0028-6 PMC5742028

[R73] Zhang Y.-D., Dong Z., Wang S.-H., Yu X., Yao X., Zhou Q., Hu H., Li M., Jiménez-Mesa C., Ramirez J., (2020). Advances in multimodal data fusion in neuroimaging: Overview, challenges, and novel orientation. Information Fusion, 64, 149–187. 32834795 10.1016/j.inffus.2020.07.006 PMC7366126

[R74] Zhao N., Yuan L.-X., Jia X.-Z., Zhou X.-F., Deng X.-P., He H.-J., Zhong J., Wang J., & Zang Y.-F. (2018). Intra-and inter-scanner reliability of voxel-wise whole-brain analytic metrics for resting state fmri. Frontiers in neuroinformatics, 12, 54. 30186131 10.3389/fninf.2018.00054 PMC6110941

